# Hydrogen sulphide reduced the accumulation of lipid droplets in cardiac tissues of db/db mice via Hrd1 S‐sulfhydration

**DOI:** 10.1111/jcmm.16781

**Published:** 2021-09-25

**Authors:** Yu Sun, Linxue Zhang, Baoling Lu, Jingchen Wen, Mengyi Wang, Shiwu Zhang, Qianzhu Li, Feng Shu, Fangping Lu, Ning Liu, Shuo Peng, Yajun Zhao, Shiyun Dong, Fanghao Lu, Weihua Zhang, Yan Wang

**Affiliations:** ^1^ Department of Pathophysiology Harbin Medical University Harbin China; ^2^ Department of Infectious The Fourth Hospital of Harbin Medical University Harbin China; ^3^ Department of Urologic Surgery First Affiliated Hospital of Harbin Medical University Harbin China

**Keywords:** DGAT, diabetic cardiomyopathy, Hrd1, hydrogen sulphide, Lipid droplets, S‐sulfhydration

## Abstract

Accumulation of lipid droplets (LDs) induces cardiac dysfunctions in type 2 diabetes patients. Recent studies have shown that hydrogen sulphide (H_2_S) ameliorates cardiac functions in db/db mice, but its regulation on the formation of LDs in cardiac tissues is unclear. Db/db mice were injected with NaHS (40 μmol·kg^‐1^) for twelve weeks. H9c2 cells were treated with high glucose (40 mmol/L), oleate (200 µmol/L), palmitate (200 µmol/L) and NaHS (100 µmol/L) for 48 hours. Plasmids for the overexpression of wild‐type Hrd1 and Hrd1 mutated at Cys115 were constructed. The interaction between Hrd1 and DGAT1 and DGAT2, the ubiquitylation level of DGAT1 and 2, the S‐sulfhydration of Hrd1 were measured. Exogenous H_2_S ameliorated the cardiac functions, decreased ER stress and reduced the number of LDs in db/db mice. Exogenous H_2_S could elevate the ubiquitination level of DGAT 1 and 2 and increased the expression of Hrd1 in cardiac tissues of db/db mice. The S‐sulfhydration of Hrd1 by NaHS enhanced the interaction between Hrd1 and DGAT1 and 2 to inhibit the formation of LD. Our findings suggested that H_2_S modified Hrd1 S‐sulfhydration at Cys115 to reduce the accumulation of LDs in cardiac tissues of db/db mice.

## 1 INTRODUCTION

The prevalence of obesity and diabetes is rapidly growing worldwide due to diet and lifestyle and the increase in the ageing of population.^1^, ^2^ Patients suffering from diabetes are more likely to develop cardiovascular diseases than those without diabetes, and mounting evidence demonstrates that diabetes is an independent factor leading to cardiac structural and functional abnormality.^3^ Diabetic cardiomyopathy (DCM) is defined as ventricular hypertrophy and contractile dysfunctions without other cardiac diseases, such as coronary artery disease and hypertension,^4^, ^5^ but the pathophysiological mechanisms on DCM are unclear. Hyperglycaemia and hyperlipidaemia are characterized as type 2 diabetes. The heart is exposed to high concentration of fatty acids, which exceed those used by the heart under physiological condition.^6^ This overdose of fatty acids enters the cardiomyocytes and thereby promotes the formation of lipid droplets (LDs).^7^ A great amount of LDs in the heart is one of the hallmarks of DCM.^8^ Nascent LD formation is believed to originate from the accumulation of triglyceride (TAG) between the two leaflets of the endoplasmic reticulum (ER) and is released into cytosol.^9^ The ER plays a crucial role in the synthesis and storage of protein and lipid.^10^ ER homeostasis can be disturbed by dysfunction of LD biogenesis, which causes ER stress to trigger unfolded protein response (UPR).^11^ The UPR is an adaptive mechanism that leads to reduce protein translation, increase transcription of gene related to ER stress and enhance ER‐associated protein degradation (ERAD).^12^ Hrd1 is an ER‐transmembrane E3 ubiquitin ligase, and mounting evidence reveals that this enzyme plays a crucial role in the ERAD of misfolded proteins.^13^ Some studies have confirmed that Hrd1 contributed to the adaptive ER stress response, which preserves cardiac function in mouse model of pathological cardiac hypertrophy.^14^ Due to LDs originating from ER, dysfunctional LDs can induce ER stress.^15^ Recent studies on the formation and biological function of LDs have mainly focussed on adipose and liver,^16^ but few studies have investigated the mechanisms in Hrd1 modulating the formation of LDs in the cardiovascular system.

Hydrogen sulphide (H_2_S) is considered the third gasotransmitter, after nitric oxide and carbon monoxide. Endogenous H_2_S is produced via catalysed reactions by three enzymes in mammalian cells: cystathionine‐β‐synthase (CBS), cystathionine‐γ‐synthase (CSE) and 3‐mercaptopyruvate sulphurtransferase (3‐MST).^17^ H_2_S plays a key regulatory role in cardiovascular homeostasis that scavenges reactive oxygen species (ROS), reduces apoptotic signalling, modulates mitochondrial respiration and decreases inflammation.^18^ Furthermore, H_2_S exerts metabolic regulation effects on hearts.^19^, ^20^ Increasing evidence suggests that the circulating levels of H_2_S are decreased in animal models of diabetes and in T2DM patients.^21^, ^22^ NaHS and Na_2_S are two well‐used H_2_S donor and convenient to handle; however, H_2_S is released immediately from NaHS and Na_2_S in biological media. Previous studies have been reported that NaHS have an immediate but long‐lasting effect via protein sulfhydration or altering gene expression.^23^, ^24^ In the present study, we used a well‐established type 2 diabetes model (db/db mice) to investigate the mechanism through which exogenous H_2_S regulates the formation of LDs.

## 2 METHODS

### 2.1 Experimental animals

Homozygous male and female ten‐week‐old db/db mice on a C57BL/6 background (n = 60) and their corresponding wild‐type (n = 30) littermates were used in this study. All mice were provided by the Animal Laboratory Centre of Nanjing University. Animals were housed in a climate‐ and temperature‐controlled room, on a 12:12 hours light‐dark cycle. The mice were maintained on a standard diet and water ad libitum. Half of the db/db mice were put in the NaHS treatment group and treated with NaHS (40 μmol·kg^‐1^) by intraperitoneal injection every 2 days for twelve weeks. All animal experiments were performed according to the Guide for the Care and Use of Laboratory Animals published by the China National Institutes of Health and approved by the Animal Care Committees of Harbin Medical University, China.

### 2.2 Echocardiographic analysis of cardiac function

Mice were sedated with avertin at a dose of 240 mg·kg^‐1^ and placed in the supine position. Two‐dimensionally guided M‐mode recordings were obtained from the short‐axis view at the level of the papillary muscles by using either an Acuson Sequoia system and an Acuson 15‐MHz linear‐array transducer or a GE Vivid 7 system with a GE S10‐MHz phased‐array transducer (General Electric). Left ventricular parameters were measured including ejection fraction (EF, %) and fractional shortening (FS, %).

### 2.3 Transmission electron microscopy assay

Ultrastructural alterations in cardiac tissues were detected by transmission electron microscopy (TEM). Cardiac tissues for TEM were cut into pieces less than 1 mm3 and fixed in 2.5% glutaraldehyde in 0.1 mol/L sodium cacodylate buffer (pH 7.4) for 4 hours. Tissues were post‐fixed in osmium tetroxide and embedded in Epon 812(Electron Microscopy Sciences). Ultrathin sections were stained with uranyl acetate and lead citrate and examined under a Zeiss Axiophot microscope.

### 2.4 Measurement of hydrogen sulphide level

The measurement of H_2_S level in plasma and isolated cardiac tissues followed the previously established protocol.^25^ Briefly, cardiac tissues were homogenized in a 50 mmol/L ice‐cold potassium phosphate buffer (pH 6.8) containing a 100 mmol/L potassium phosphate buffer, 10 mmol/L L‐cysteine and 2 mmol/L pyridoxal 5′‐phosphate. Plasma and cardiac sample were mixed with 10% trichloroacetic acid, respectively. The reaction was stopped by 1% zinc acetate, followed by incubation with N, N‐dimethyl‐p‐phenylenediamine sulphate (DPD) for 15 minutes. The absorbance at 670 nm was measured with a spectrophotometer.

### 2.5 Cellular experimental protocol

The cultured H9c2 were randomly divided into the following groups and treatments: control group (low glucose, LG, 5.5 mmol/L), high glucose (HG, 40 mmol/L) +Oleate (Ole, 200 µmol/L)+ Palmitate (Pal, 200 µmol/L), HG+Ole+Pal+NaHS (sodium hydrosulphide, 100 µmol/L), HG+Ole+ Pal+PYR41 (50 µmol/L, an inhibitor of ubiquitin‐activating enzyme), HG+Ole+Pal+MG132 (20 µmol/L, an inhibitor of proteasome), HG+Ole+ Pal+4‐PBA (4‐phenyl butyric acid, 5 mmol/L, an inhibitor of endoplasmic reticulum stress), HG+Ole+Pal+Tg (thapsigargin, 100 µmol/L, an inducer of endoplasmic reticulum stress), HG+Ole+Pal+PPG (DL‐propargylglycine, 10 nmol/L, an irreversible competitive CSE inhibitor), HG+Ole+Pal+DTT (dithiothreitol, 20 µmol/L, an inhibitor of disulphide bond). Drugs were added directly in cultured medium for 48 hours. The H9c2 cells were treated with high glucose and palmitate and oleate classically mimic hyperglycemia and hyperlipidemia.

### 2.6 Isolation of lipid droplets in cardiac tissues

Isolation of lipid droplets in cardiac tissues was prepared by previously described methods.^26^ Cardiac tissues were homogenized (100 mg cardiac tissues/1mL ice‐cold Tris‐EDTA buffer pH7.4 with cocktail inhibitors) on ice. Homogenates were centrifuged at 100 g at 4℃ for 10 min; then, the supernatant was centrifuged at 23,7020 g for 2 h at 4℃ (Ultracentrifuge, Beckman Optima). The LDs were concentrated at the top of the tube and then resuspended the pellets with 200 µL of Tris‐EDTA buffer pH 7.4 and centrifuged at 18,000g for 30 min at 4℃. Working rapidly removed the solution underlying the LDs. LDs were resuspended with 200 ml of Tris‐EDTA buffer pH 7.4. Proteins were then acetone‐precipitated from the remaining LD containing solution by adding 1:3 (v/v) of acetone at 20℃ overnight. The following day, collected the LD proteins by centrifuging the Eppendorf tubes for 1 hour at 18,000 g at 4℃.

### 2.7 BODIPY 493/503 staining

Isolated cardiac tissues from db/db mice and wild‐type mice were washed with PBS and fixed in 4% paraformaldehyde overnight before being dehydrated 30% sucrose for 48 hours. Cardiac sections were embedded into Tissue Tek OCT compound (Sakura) for histology. Embedded left ventricles were cut into 4 μm sections. The H9c2 cells were fixed with 4% paraformaldehyde for 20 min. Frozen cardiac tissue or H9c2 cells immersed in BODIPY 493/503 solution for 30 min at 37℃. After the samples were washed 3 times with PBS, the stained droplets were observed using a fluorescence microscope (Olympus, XSZ‐D2, Japan).

### 2.8 BODIPY 558/568 C12 staining

H9c2 cells were incubated with 2 µmol/L BODIPY 558/568 C12 (Life technologies) and treated with different reagents after 48 hours. Cells were washed three times with DMEM. BODIPY 558/568 C12 fluorescence was determined using fluorescence microscope (Olympus, XSZ‐D2).

### 2.9 Oil red O staining

The H9c2 cells were fixed with 4% paraformaldehyde for 20 minutes, and added Oil red O solution for 20 minutes, followed by a 60% isopropanol wash, staining with haematoxylin solution for 1 minutes and washed with water. Lipid droplets were visualized using the microscope (Olympus, XSZ‐D2).

### 2.10 LC‐MS/MS analysis

Samples were lysed and trypsin digested according to our previous procedure.^27^ The tryptic peptides were dissolved in 0.1% formic acid (solvent A), directly loaded onto a home‐made reversed‐phase analytical column (15‐cm length, 75 μm i.d.). The gradient was comprised of an increase from 6% to 23% solvent B (0.1% formic acid in 98% acetonitrile) over 26 minutes, 23%‐35% in 8 minutes and climbing to 80% in 3 minutes then holding at 80% for the last 3 minutes, all at a constant flow rate of 400 nL/min on an EASY‐nLC 1000 UPLC system. The peptides were subjected to NSI source followed by tandem mass spectrometry (MS/MS) in Q ExactiveTM Plus (Thermo) coupled online to the UPLC. The electrospray voltage applied was 2.0 kV. The m/z scan range was 350‐1800 for full scan, and intact peptides were detected in the Orbitrap at a resolution of 70,000. Peptides were then selected for MS/MS using NCE setting as 28 and the fragments were detected in the Orbitrap at a resolution of 17 500. A data‐dependent procedure that alternated between one MS scan followed by 20 MS/MS scans with 15.0s dynamic exclusion. Automatic gain control (AGC) was set at 5E4. The parameter settings for mass spectrometer were referred to our published reports.

### 2.11 Protein identification and quantification

The resulting MS/MS data were processed using MaxQuant search engine (v.1.5.2.8). Tandem mass spectra were searched against human UniProt database concatenated with reverse decoy database. Trypsin/P was specified as cleavage enzyme allowing up to 4 missing cleavages. The mass tolerance for precursor ions was set as 20 ppm in First search and 5 ppm in Main search, and the mass tolerance for fragment ions was set as 0.02 Da. The false discovery rate (FDR) was adjusted to <1%, and minimum score for modified peptides was set >40. The mass tolerance for fragment ions was set to 0.5 Da. For label‐free quantification, protein expression levels were estimated using the iBAQ (Intensity Based Absolute Quantification) algorithm embedded in MaxQuant.^28^ In brief, protein expression level was calculated by the sum of peak intensities (normalized by the number of theoretically observable peptides) of all peptides matching to the corresponding protein. The value of iBAQ is proportional to the relative expression level of protein. We further normalized the expression levels of each sample by dividing each raw iBAQ value by the median value.

### 2.12 Bioinformatic analysis

A volcano plot was constructed to better visualize and identify the differentially expressed proteins between groups. Hierarchical clustering analysis was carried out using Cluster 3.0 software,^29^ and a heat map was produced accompanied by a dendrogram depicting the extent of similarity of protein expression among the samples. For the convenience of gene annotation, corresponding Ensembl gene IDs of the differentially expressed proteins were used for further bioinformatics analysis. To characterize these genes, we tested them for enrichment of gene ontology (GO) biological process, cellular component and molecular function terms by using DAVIDs Functional Annotation Chart tool (Version 6.8). A *P* value < .05 was controlled for significant enrichment. An important portion of enriched GO terms was selected to construct a network with related proteins using Cytoscape.

### 2.13 Immunoblot analysis

Western blotting was performed as described previously. Primary antibodies included anti‐CSE (Proteintech,1:1000), anti‐CHOP (Proteintech, 1:1000), anti‐Ubiquitin (Proteintech, 1:1000), anti‐eIF2α (Proteintech, 1:1000), anti‐P‐eIF2α (Ser51, Cell Signaling Technology, 1:1000), anti‐DGAT1 (Proteintech, 1:1000), anti‐DGAT2(Signalway Antibody, 1:1000), anti‐PERK (Proteintech, 1:1000), anti‐P‐PERK (Thr‐980, Cell Signaling Technology, 1:1000), anti‐Bip (Proteintech, 1:1000), anti‐Hrd1 (Proteintech, 1:1000), anti‐perilipin2 (Proteintech, 1:1000), anti‐collagen I (Proteintech, 1:1000), anti‐collagen Ⅲ (Proteintech, 1:1000), anti‐GAPDH (Proteintech, 1:1000). Primary antibodies were incubated overnight at 4℃. Densitometry was conducted with image processing and analysis program AlphaView.SA and the data were expressed as relative units.

### 2.14 Immunoprecipitation

The cardiac tissues and H9c2 cells were harvested and lysed as previously described.^30^ Briefly, cardiac tissues or H9c2 cells lysates were diluted at 2 mg/mL. About 500 μg per sample of protein was used for immunoprecipitation. Protein A/G magnetic beads for immunoprecipitation were conjugated with antibody (10 μg antibody per 500 μg protein) and incubated with H9c2 cells or cardiac tissues lysates overnight at 4℃ with gentle rotation. Beads were collected using centrifugation at 4℃, 10 000 *g* for 5 minutes, and beads were washed with cell lysis buffer containing 1% PMSF, three times. The precipitates were diluted with loading buffer and boiled for 10 minutes at 100℃ and later used for Western blotting analyses to detect potential interacting proteins.

### 2.15 S‐sulfhydration assay

The assay was carried out as described previously.^31^ Briefly, cardiac tissues and cells were homogenized in HEN buffer [250 mmol/L Hepes‐NaOH (pH 7.7), 1 mmol/L EDTA, and 0.1 mmol/L neocuproine] supplemented with 100 μmol/L deferoxamine and centrifuged at 13 000 *g* for 30 minutes at 4℃. Lysates (240 μg) were added to blocking buffer (HEN buffer adjusted to 2.5% SDS and 20 mmol/L MMTS) at 50℃ for 20 minutes with frequent vortexing. The MMTS was then removed by acetone, and the proteins were precipitated at −20℃ for 20 minutes. After acetone removal, the proteins were resuspended in HENS buffer (HEN buffer adjusted to 1% SDS). To the suspension was added 4 mmol/L biotin‐HPDP in dimethyl sulphoxide without ascorbic acid. After incubation for 3h at 25℃, biotinylated proteins were precipitated by streptavidin‐agarose beads, which were then washed with HENS buffer. The biotinylated proteins were eluted by SDS‐polyacrylamide gel electrophoresis (SDS‐PAGE) sample buffer and subjected to Western blot analysis.

### 2.16 Point mutation of Hrd1

Adenoviruses expression GFP and Hrd1‐GFP were purchased from Cyagen Biosciences Inc. The full‐length mouse Hrd1 with a single mutation of cysteine 115 to alanine and GFP cDNA was inserted into pM vector (Cyagen Biosciences) between the Kozak and T2A sites. The adenovirus was added directly to H9c2 cells and after 4‐6h for transfection, new fresh medium was added. The H9c2 cells were treated with different reagents after 48h, and the related proteins were detected by Western blot.

### 2.17 Overexpression of Hrd1 plasmid construction

Adenoviruses expression Hrd1‐FLAG were purchased from Cyagen Biosciences Inc. To construct plasmid expression Hrd1‐flag proteins, the mouse Hrd1 gene was cloned into pM vector between the Kozak and T2A sites. The adenovirus was added directly to H9c2 cells and after 4‐6h for transfection, new fresh medium was added. The H9c2 cells were treated with different reagents after 48 hours, and the related proteins were detected by Western blot.

### 2.18 Statistical analysis

Results were analysed by using the Prism software package (GraphPad Software). Results are expressed as the mean ± standard error (SEM). More than two groups were compared using a one‐way ANOVA and Bonferroni's correction. Differences between individual groups were analysed using Student's *t* test.

## 3 RESULTS

### 3.1 Characteristics of db/db mice

Db/db mice, leptin receptor deficiency, are widely used as an animal model of type 2 diabetes. We first examined the parameters of the main characteristics of db/db mice. As shown in Figure S1A‐E, the blood glucose level and the plasma concentrations of TAG, FFA and insulin were significantly higher in db/db mice than in the wild‐type mice and the NaHS‐treated wild‐type mice. To detect collagen deposition in cardiac tissues, we tested the protein level of collagen I, collagen Ⅲ. Our results found that exogenous H_2_S reduced the expression of collagen I, collagen Ⅲ in db/db mice (Figure S1F,G). Our data showed that the area of cardiomyocytes in db/db mice was obviously larger than that in wild group and db/db+NaHS groups (Figure S1H). These results showed that db/db mice can be used as a typical type 2 diabetic animal model.

### 3.2 H_2_S level and CSE expression in cardiomyocytes in hyperglycaemic and hyperlipidaemic state

**FIGURE 1:**
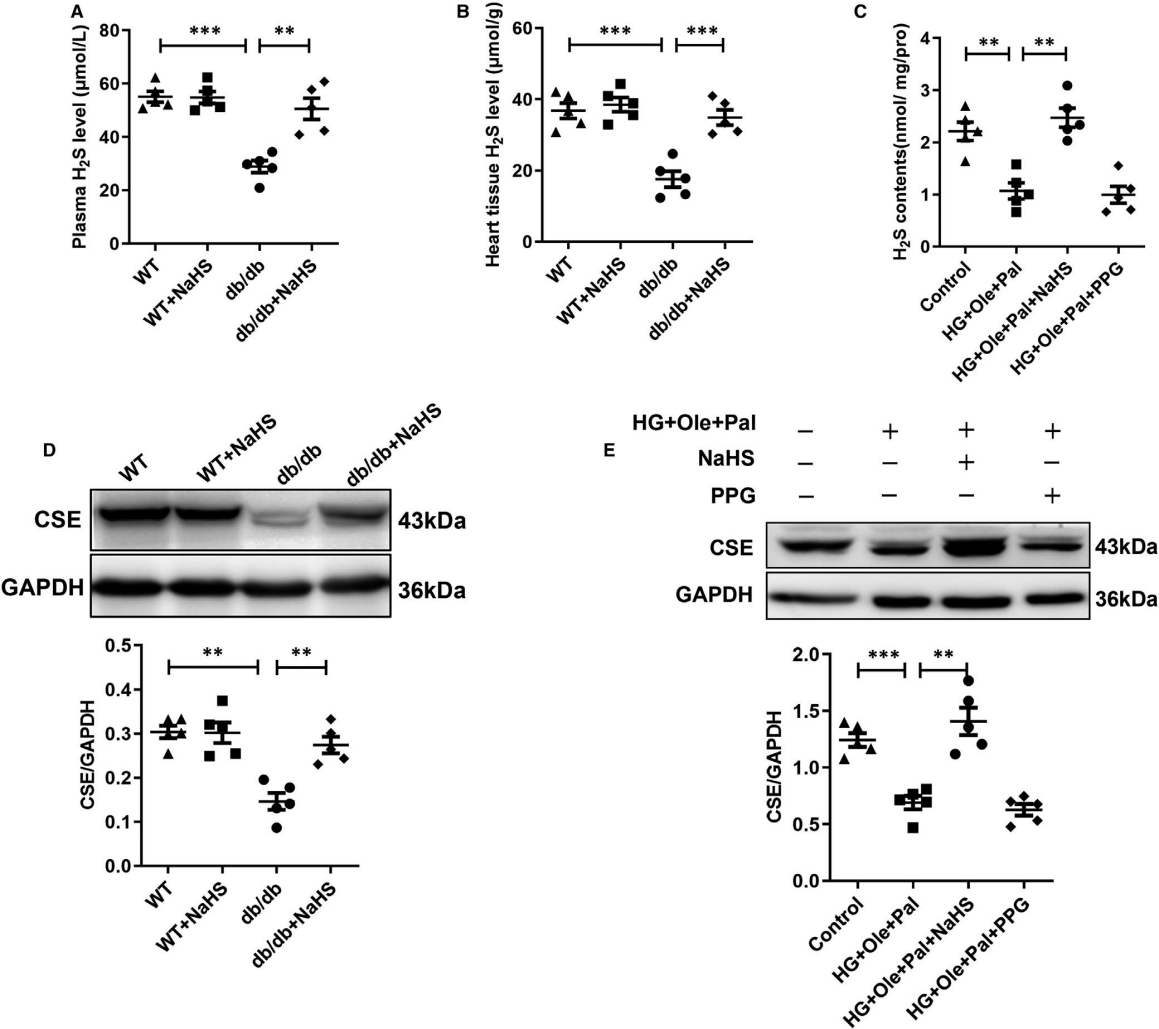
The H_2_S level and CSE expression. A, Plasma H_2_S level in cardiac tissues. B, Cardiac H_2_S levels. C, H_2_S level in cardiomyocytes. D, CSE expression in cardiac tissues. E, CSE expression in cardiomyocytes. The values represent the means ± SE, *P < .05, **P < .01, ***P < .001, n = 5

An increasing number of studies have suggested that H_2_S is an important gasotransmitter generated by CSE in the cardiovascular system.^32^ In the present study, we assessed the H_2_S level and CSE expression in cardiac tissues in db/db mice and in H9c2 cells. Our results showed that the H_2_S level in plasma and cardiac tissues of db/db mice was significantly lower than that of those in wild‐type, wild‐type and db/db mice with treatment of NaHS (Figure 1A,B). H9c2 cells were treated with 40 mmol/L glucose, 200 µmol/L palmitate and 200 µmol/L oleate to obtain a cellular model that mimics type 2 diabetes. The H_2_S level in the H9c2 cells in the HG+Ole+Pal group was also decreased compared with those in the control and NaHS‐treated H9c2 cells (Figure 1C). Additionally, the expression of CSE in cardiac tissues of db/db mice was decreased compared with those in wild‐type and wild‐type and db/db with treated by NaHS groups (Figure 1D). Our previous study showed that exogenous H_2_S reduced the ubiquitylation level of CSE to inhibit the degradation of CSE.^21^ Similarly, the expression levels of CSE in the groups treated with HG+Ole+Pal and PPG (an inhibitor of CSE) were also lower than those in the control and NaHS groups (Figure 1E). These results suggested that the endogenous H_2_S level is reduced under hyperglycaemic and hyperlipidaemic state.

### 3.3 Exogenous H_2_S ameliorated DCM in db/db mice

**FIGURE 2:**
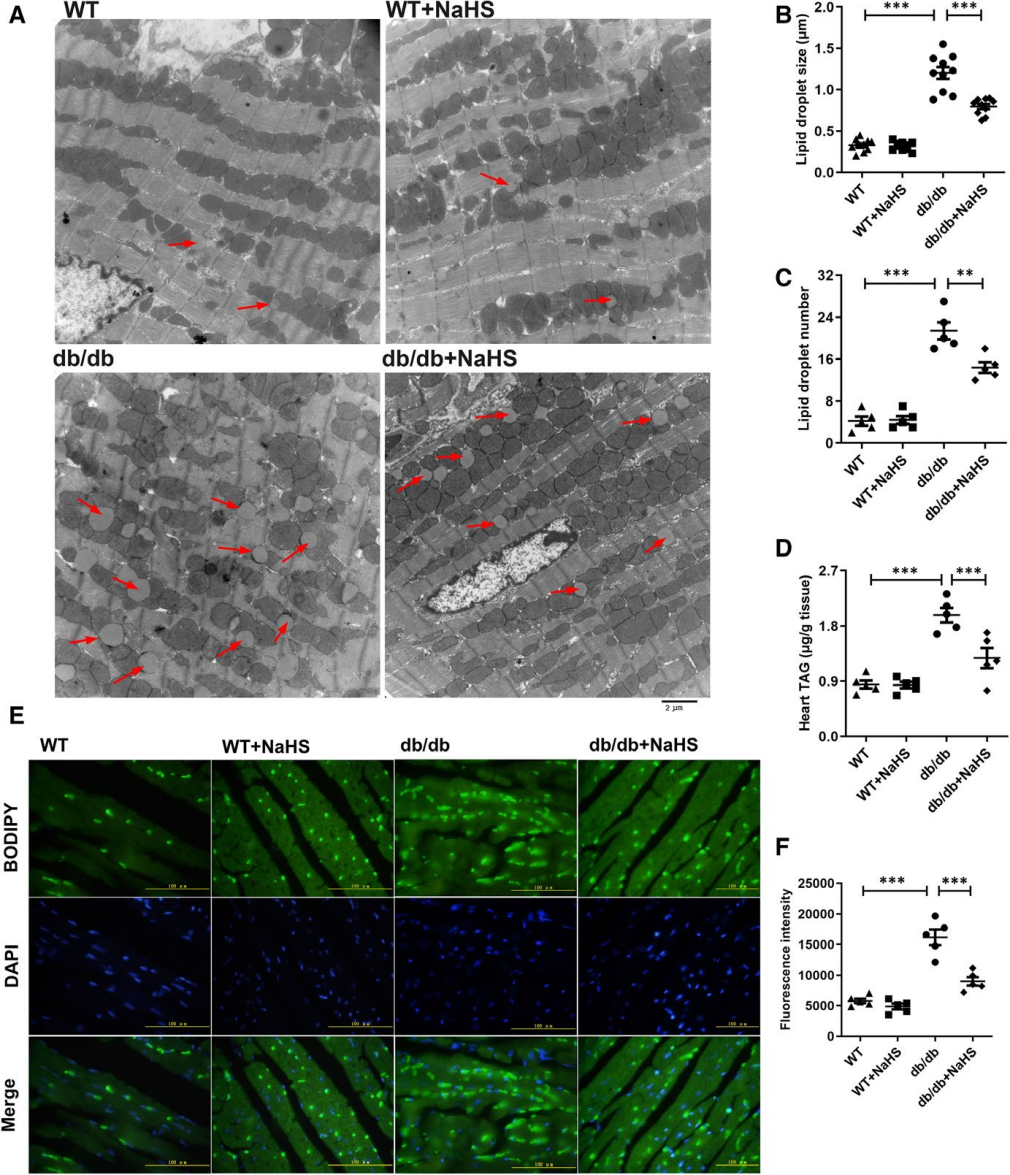
Exogenous H_2_S reduces the number and size of lipid droplets in the cardiac tissues of db/db mice. A, Transmission electron microscopy of the cardiac tissues in db/db mice. Lipid droplets were indicated by red arrows. B, Lipid droplet size (n = 10). C, Lipid droplet number (n = 5). D, Cardiac triglyceride content(n = 5). E, The droplets in cardiac tissues of db/db mice detected by the fluorescence probe BODIPY 493/503. F, Quantification of fluorescence intensity statistics(n = 5). The values represent the means ± SE. **P < .01, ***P < .001

To further investigate the effects of H_2_S on cardiac functions, the LVEF, LVFS, LVED, LV mass and heart rate were analysed. The LVEF, LVFS and LVED values were decreased in db/db mice and improved by NaHS treatment. However, the LV mass and left ventricular end‐diastolic posterior wall (LVPW(d)) in db/db mice were significantly higher than that in the NaHS‐treated db/db mice (Table 1 and Figure S2A). LVEF and LVFS are dependent on heart rate; our results showed the reduction in heart rate in db/db mice compared with wild‐type mice. Heart size of db/db mice was visibly and hypertrophic as demonstrated by the increased left ventricular weight/ tibia length ratio indexes compared to wild‐type mice (Table 1). The ultrastructural morphology of cardiac tissues was observed by transmission electron microscopy (TEM), and almost no LDs were found in the cardiac tissues of the control and NaHS‐treated control mice (Figure S2B). The TEM observations also indicated that the size and number of LDs in cardiac tissues of db/db mice were increased in a time‐dependent manner, whereas exogenous H_2_S significantly reduced the number and size of LDs in cardiac tissues (Figure 2A‐C). As shown in Figure 2E, the staining of neutral fat with BODIPY 493/503 also confirmed that the number of droplets was abundant and TAG content in cardiac tissues of db/db mice was obviously higher than those in wild‐type and db/db mice with treatment of NaHS (Figure 2D). To further investigate whether exogenous H_2_S affected the formation of LDs in H9c2 cells in time‐dependent manner, we utilized the BODIPY 558/568 (Red C12), which is a saturated FA analog with a tail containing 12 carbons and BODIPY 558/568 fluorophore covalently bound at the hydrophobic end, which is shown to incorporate into LD‐specific neutral lipids, and BODIPY 493/503 probe to detect the number of LDs.^33^ The number of LDs in the HG+Ole+Pal group increased in time‐dependent manner compared with that in the NaHS‐treated group (Figure S3). These results demonstrated that exogenous H_2_S reduced the number of LDs under hyperglycaemic and hyperlipidaemic state.

**TABLE 1:** Cardiac function of mice was examined by heart echocardiography

	WT	WT+NaHS	db/db	db/db+NaHS
LVEF (%)	77.94 ± 5.88	79.29 ± 7.22	61.92 ± 4.77*	74.53 ± 4.90^#^
LVFS (%)	49.47 ± 5.81	46.66 ± 5.29	32.06 ± 5.60**	44.92 ± 4.48^##^
LVED Vol (μL)	50.91 ± 8.54	48.22 ± 4.29	28.98 ± 6.45**	45.53 ± 6.52^##^
LV Mass(mg)	76.76 ± 8.72	76.43 ± 7.19	127.93 ± 19.77***	107.81 ± 14.91^#^
LVPW(d)(mm)	0.73 ± 0.060	0.72 ± 0.11	1.17 ± 0.18***	0.90 ± 0.12^#^
LV weight/Tibia length(mg/mm)	3.31 ± 0.26	3.27 ± 0.30	5.10 ± 0.37***	4.34 ± 0.47^##^
Heart rate (bpm)	462 ± 10	458 ± 15	379 ± 20*	395 ± 18

Note

The values represent the means ± SE, n = 5.

Abbreviations: LVEF, left ventricular end‐diastolic volume; LV Mass, left ventricular mass; LVED Vol; LVEF, left ventricular ejection fraction; LVFS, left ventricular fractional shortening; LVPW(d), left ventricular end‐diastolic posterior wall.

*P < 0 .05, **P < 0.01, ***P < .001 vs WT group, ^#^ *P* < .05, ^##^ *P* < .01 vs db/db group.

### 3.4 Exogenous H_2_S inhibited LD formation through attenuating ER stress

Some studies have demonstrated that excess saturated free fatty acids (sFAs) could trigger unfold protein response (UPR) and thereby lead to ER stress.^34^ The formation of LDs was initiated by the occurrence of ER stress.^35^ In this study, the expression of Bip, CHOP, p‐eIF2α/ eIF2α, p‐PERK/PERK, which are the hallmarks of ER stress, was significantly increased in cardiac tissues of db/db mice compared with those of wild‐type and db/db mice treated with NaHS (Figure S4A). In addition, the expression level of the above‐mentioned proteins in the cells treated with HG+Ole+Pal and Tg (an ER stress inducer and SR/ER Ca^2+^‐ATPase inhibitor) was significantly elevated compared with those in the control cells and the cells treated with NaHS and 4‐PBA (an inhibitor of ER stress) (Figure S4B).

To further investigate whether ER stress promotes LD formation, the fluorescent probe BODIPY 558/568 (Red C12) was used to detect the accumulation of LDs in H9c2 cells. Due to the synthesis of neutral lipids in the outer and inner membranes of the ER, the location of LD formation was examined using an ER tracker. Our results showed that the number of LDs in the HG+Ole+Pal and Tg groups was markedly greater than that in the control, NaHS and 4‐PBA groups (Figure S4C and D ). These data suggested that LD formation was closely associated with the induction of ER stress in response to hyperglycaemic and hyperlipidaemic state.

### 3.5 Comparative proteomic analysis of cardiac tissues from db/db and NaHS‐treated db/db mice

Substantial evidence indicates that the formation of LDs is catalysed by complicated enzyme systems, which are elaborated in liver, however, it is indistinct in heart. High‐through proteomics technologies combined with bioinformatics approaches have been applied for the identification of proteins associated with the cellular events that contribute to diseases. In this study, a liquid chromatography‐tandem mass spectrometry (LC‐MS) analysis of cardiac tissues from db/db and db/db mice treated with NaHS was performed.

**FIGURE 3:**
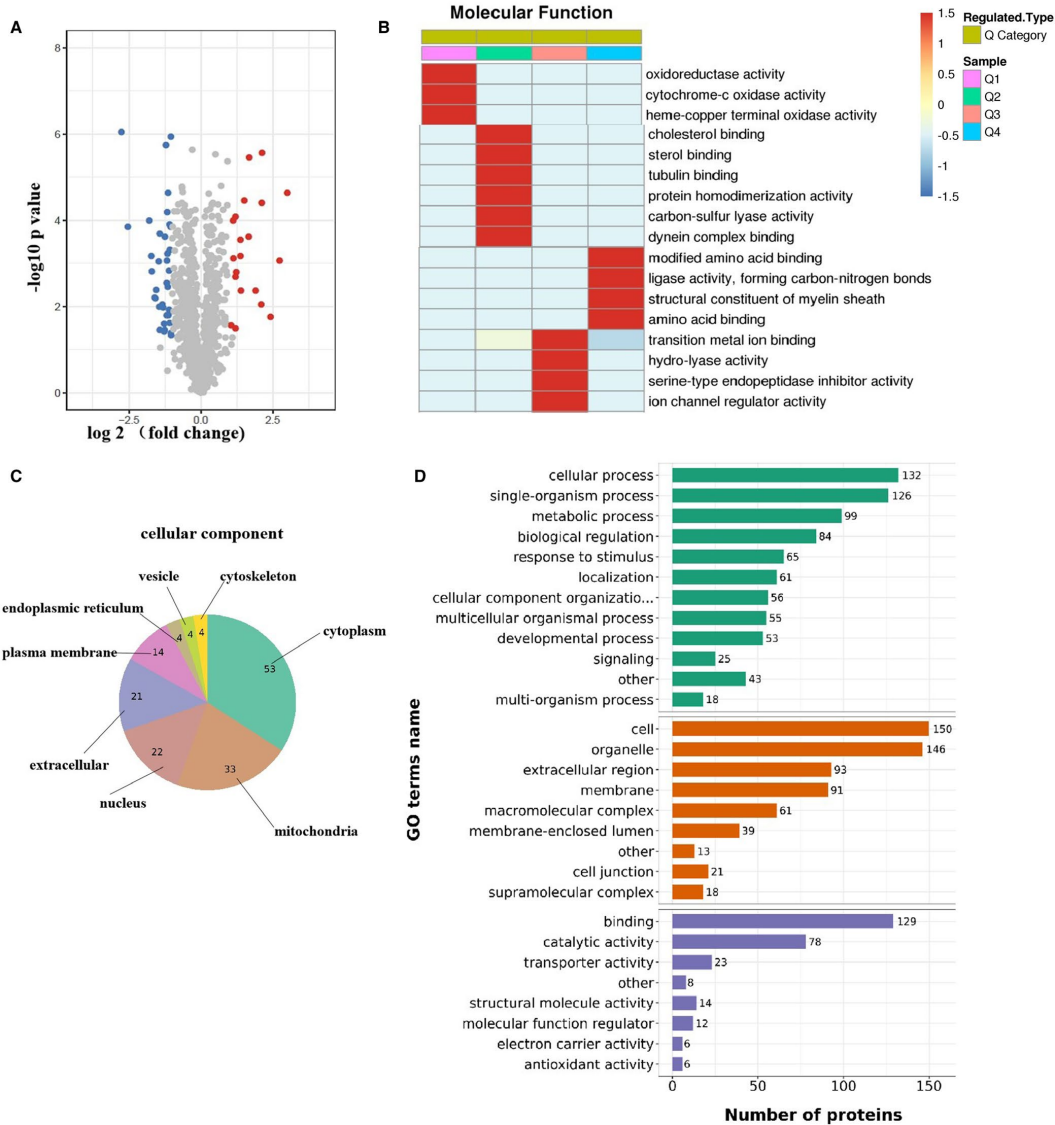
Proteomic analysis of cardiac tissues from db/db mice and with the treatment of NaHS. A, Volcano plot showing the quantitative protein expression in cardiac tissues from db/db mice and NaHS‐treated db/db mice. Proteins showing differential expression with fold change >1.5 are marked in colour. B, Heatmap of the molecular functions of the differentially expressed proteins between db/db mice and NaHS‐treated db/db mice (Q means the ratio of db/db‐NaHS group to db/db, Q1 < 0.5, Q2 = 0.5‐0.66, Q3 = 1.5‐2, Q4 > 2; red indicates strong functional enrichment, and the blue colour shows weak functional enrichment). C, Subcellular distribution of the differentially expressed proteins. D, Gene ontology between db/db mice and NaHS‐treated db/db mice

A total of 2368 proteins were identified, and 1403 proteins were quantified in cardiac tissues. Using the criteria fold change >1.5 and *P* value < .05, 156 proteins were identified as differentially expressed (DE) between groups (Table S1). Among these, 55 proteins were up‐regulated and 101 proteins were down regulated in cardiac tissues in db/db mice treated by NaHS compared with db/db mice (Figure 3A). To obtain an overview of the function of DE proteins, a molecular function enrichment analysis was conducted (Figure 3B), and the analysis showed enrichment of the molecular functions related to oxidoreductase activity, cytochrome c activity, sterol binding and ligase activity in the cardiac tissues of NaHS‐treated db/db mice compared with db/db mice. The subcellular location of DE proteins was annotated using GO using a database. As shown in Figure 3C, the largest proportion of DE proteins was identified in cytoplasma (53), followed by mitochondria (33), nuclei (22), extracellular (21), plasma membrane (14). The major GO terms including the related biological processes were identified. DE proteins were found to be enriched in metabolic processes, myosin filament organization, mitochondria fission and respiration process (Figure 3D).

Among these DE proteins, HMG‐CoA reductase degradation protein 1(Hrd1, fold change of 4.308, *P* = 0.00902) is of great interest because Hrd1 is the core structural component of a large endoplasmic reticulum membrane‐anchored protein complex and plays a role as an E3 ligase for the degradation of luminal and membrane substrates. Maladaptive ER stress increases the degradation of misfolded proteins by ERAD. Hrd1 plays a key role in the ERAD of a wide spectrum of proteins. Therefore, we focussed on the function of Hrd1 in cardiac tissues of db/db and NaHS‐treated db/db mice.

### 3.6 Exogenous H_2_S regulated the interaction with DGATs and Hrd1

**FIGURE 4:**
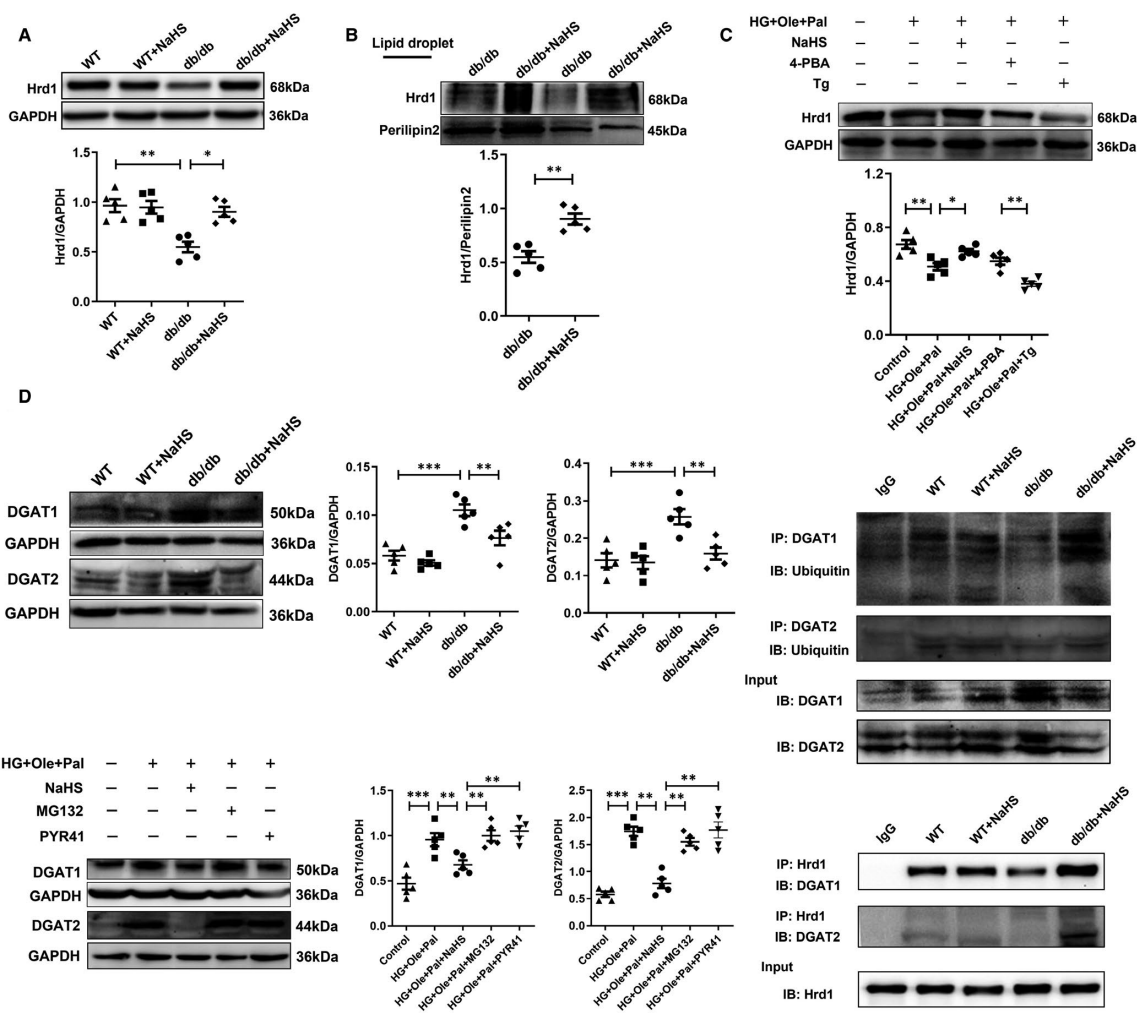
Exogenous H_2_S regulates the interaction between DGAT and Hrd1. A, Expression of Hrd1 in cardiac tissues. B, Expression of Hrd1 in lipid droplet in cardiac tissues (C) Expression of Hrd1 in H9c2 cells under hyperglycemic and hyperlipidemic conditions. D, Expression of DGAT1 and DGAT2 in cardiac tissues. E, Ubiquitination level of DGAT1 and DGAT2 in cardiac tissues. F, Expression of DGAT1 and DGAT2 in H9c2 cells under hyperglycaemic and hyperlipidaemic conditions. G, The interaction between Hrd1 and DGAT1 and the interaction between Hrd1 and DGAT2 were detected by coimmunoprecipitation. The values represent the means ±SE, *P <.05, **P <.01, ***P <.001, n = 5

We subsequently tested the expression of Hrd1 to validate the results from the proteomic analysis and found that the expression of Hrd1 was significantly decreased in cardiac tissues and LDs extracted from the hearts of db/db mice compared with those obtained from the wild‐type and NaHS‐treated groups (Figure 4A,B). The alteration of Hrd1 expression in the cellular model was similar to that in the animal model (Figure 4C), and these results were consistent with LC‐MS/MS analysis.

To investigate the involvement of Hrd1 in the formation of LDs, we detected the expression of DGAT1 and 2, which are key enzymes that catalyse TAG synthesis. The expression of DAGT1 and 2 in cardiac tissues of db/db mice was significantly higher than that in NaHS‐treated db/db mice (Figure 4D). To explore the causes for the down‐regulation of the expression of DGAT1 and 2 after NaHS treatment, the ubiquitylation levels of DGAT1 and 2 were measured. Our coimmunoprecipitation (Co‐IP) results showed that the ubiquitylation levels of DGAT1 and 2 was significantly increased in cardiac tissue of db/db mice after NaHS treatment compared with that of db/db mice (Figure 4E). Additionally, PYR41, an inhibitor of ubiquitin‐activating enzyme (E1), and MG132, a 26S proteasome inhibitor, were used, and the PYR41 and MG132 treatments increased the protein levels of DGAT1 and 2 in H9c2 cells (Figure 4F). Moreover, the Co‐IP results confirmed the interaction between Hrd1 and DGAT1 and 2 (Figure 4G).

**FIGURE 5:**
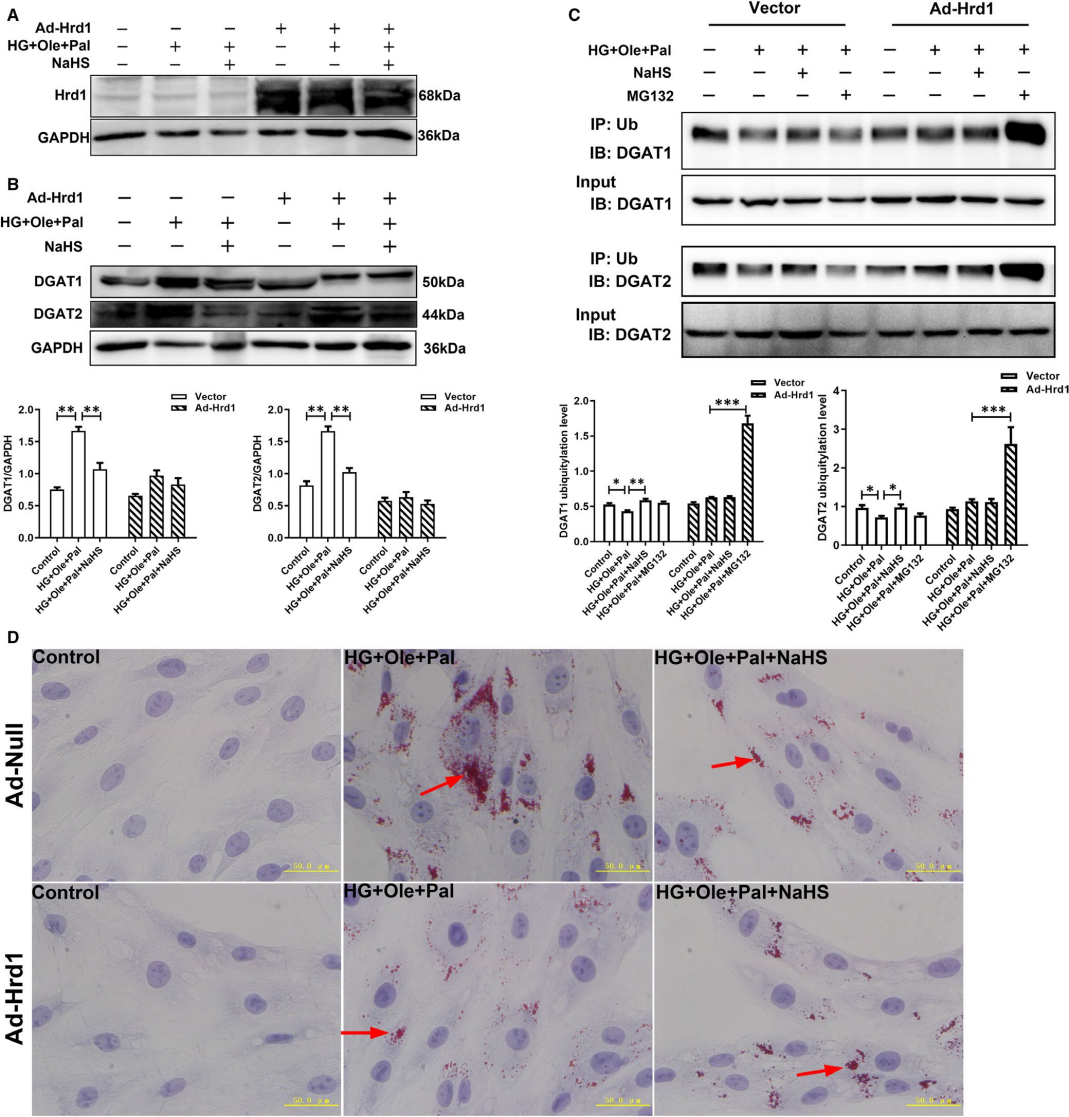
Effect of Hrd1 overexpression on the regulation of lipid droplets. A, Detection of the overexpression efficiency of Hrd1 by Western blot analysis(n = 4). B, Expression of DGAT1 and DGAT2 under treatment of overexpression of Hrd1 was detected(n = 3). C, Ubiquitination level of DGAT1 and DGAT2 after Hrd1 overexpression were detected(n = 4). D, Lipid droplets were detected by Oil red O staining. Lipid droplets were indicated by red arrows. The values represent the means ± SE. *P < .05, **P < .01, ***P < .001

To further assess whether Hrd1 modulated the ubiquitylation level of DGAT1 and 2, we constructed an Adeno‐Hrd1 expression plasmid. Western blot assay showed that the adeno‐Hrd1 plasmid‐infected H9c2 cells showed significantly increased expression of Hrd1 compared with the empty plasmid‐infected H9c2 cells (Figure 5A). Our results also revealed that overexpression of Hrd1 did not change the expression of DGAT1 and DGAT2 after HG+Ole+Pal or NaHS treatment (Figure 5B). Furthermore, we found that overexpression of Hrd1 also did not affect the ubiquitination level of DGAT1 and 2 between HG+Ole+Pal and NaHS groups, whereas the ubiquitination level of DGAT1 and 2 was significantly increased after MG132 treatment (Figure 5C). Oil red O staining analysis showed that overexpression of Hrd1 significantly decreased the number of LDs in HG+Ole+Pal group compared with the empty plasmid group (Figure 5D). These results revealed that Hrd1 increased the ubiquitylation level of DGAT1 and 2 to inhibit the formation of LDs.

### 3.7 Exogenous H_2_S regulated Hrd1 S‐sulfhydration to reduce the formation of LDs

**FIGURE 6:**
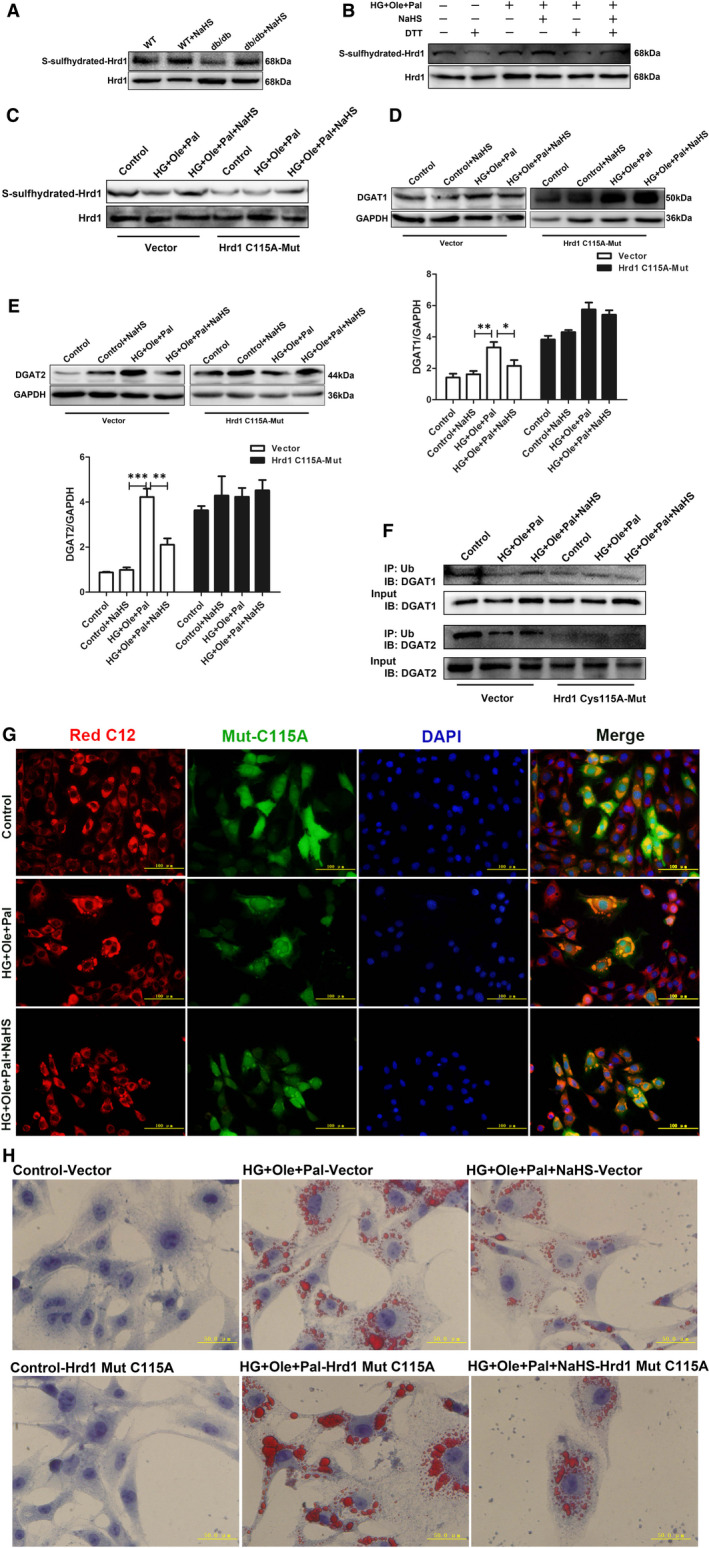
Exogenous H_2_S regulates Hrd1 S‐sulfhydration. A, The level of S‐sulfhydration of Hrd1 in cardiac tissues was detected using the biotin switch method. B, The level of S‐sulfhydration of Hrd1 in H9c2 cells under hyperglycemic and hyperlipidemic conditions was detected using the biotin switch method. C, Hrd1 mutant decreased the S‐sulfhydration level of Hrd1 in cardiomyocytes. The expression of (D) DGAT1 and (E) DGAT2 in cardiomyocytes with the Hrd1‐Cys115 mutation under hyperglycemic and hyperlipidemic conditions was detected. F, Hrd1‐Cys115 mutant affected the ubiquitination level of DGAT1 and DGAT2. G, The droplets in H9c2 cells were detected using the fluorescence probe BODIPY 558/469 C12. H, Lipid droplets H9c2 cells were detected by Oil red O staining. The values represent the means ± SE. *P < .05, **P < .01, ***P < .001, n = 5

H_2_S modifying specific cysteine residues of targeting proteins, named as polysulfidation, has been extensively studied. Protein polysulfidationas a type of post‐translational modification initiated by H_2_S could alter protein structure and activity of proteins.^36^ Our biotin switch assay showed that exogenous H_2_S enhanced the S‐sulfhydration level of Hrd1 in the hearts of WT and db/db mice treated with exogenous H_2_S (Figure 6A). In vitro, exogenous H_2_S also increased S‐sulfhydration of Hrd1, whereas 1,4‐dithiothreitol (DTT), an inhibitor of disulphide formation, reduced the S‐sulfhydration level of Hrd1 (Figure 6B). To further determine the role of Hrd1 in the degradation of DGATs, we utilized the bioinformatic methods to analyse the amino acid sequence, structure and active centre of Hrd1. Based on bioinformatic analysis, the Cys115 in active centre of Hrd1 was mutated to alaine (Figure S5A). Next, Hrd1 mutated at Cys 115 to Ala or wild type was transfected into H9c2 cells (Figure S5B). Mutant of Hrd1‐Cys115 (Hrd1‐C115A) plasmid were constructed. H_2_S could not enhance S‐sulfhydration of Hrd1 mutated at Cys115 compared with that achieved with the empty plasmid (Figure 6C). The overexpression of Hrd1‐C115A did not affect the expression of DGAT1 and 2 in H9c2 cells treated with HG+Ole+Pal and NaHS, but the protein levels of DGAT1 and 2 were significantly increased after NaHS treatment under high glucose, oleate and palmitate conditions compared with the levels obtained with the empty plasmid (Figure 6D,E). We also found that the ubiquitination level of DGAT1 and 2 at mutated Cys115 residue of Hrd1 after treatment with NaHS were decreased compared with that obtained with the empty plasmid combined with NaHS treatment (Figure 6F). The overexpression of Hrd1‐C115A increased the size and number of LDs of neutral fats stained with Oil red O and Red C12 fluorescent probe compared with those found for the empty plasmid group (Figure 6G,H). The results confirmed that exogenous H_2_S modified Hrd1 at Cys115 site to reduce the number of LDs by increasing the degradation of DGAT1 and 2.

## 4 DISCUSSION

The results from the current study show the following: (a) exogenous H_2_S decreased the number of LDs in cardiomyocytes of db/db mice; (b) exogenous H_2_S elevated the protein level of Hrd1 and the S‐sulfhydration of Cys115 in Hrd1 to enhance the interaction between Hrd1 and DGAT1 and 2 and to thereby decrease LD formation.

T2DM is associated with altered lipid metabolism, which leads to increase lipolysis and elevated fatty acid concentration in circulatory system and their enhanced uptake of fatty acids by peripheral tissues, including the heart and arteries.^37^ However, the heart uptakes large amount of fatty acids that exceed those needed for regular function, and these lipids accumulated to form LDs in cardiomyocytes, which causes cardiac steatosis.^38^ Therefore, DCM might be considered as lipid‐storage disease. Ljubkvic et al have demonstrated that the LDs accumulated in diabetic hearts and that this accumulation leaded to a significantly decreased cardiac contractile function in patients with type 2 diabetes.^39^ Left ventricular dysfunction is the earliest hallmark of diabetic cardiomyopathy, then leading to heart failure with reduced ejection fraction.^40^ Myocardial triglyceride accumulation also resulted in diastolic abnormalities. Db/db mice were characterized by progressive obese, hyperglycaemia and hyperinsulinaemia, association between altered myocardial substrate preference and cardiac dysfunction. Our results also showed that db/db mice exhibit increased accumulation of LDs in cardiac tissues and decreased cardiac function.

Increasing evidence revealed that the excessive build‐up of intracellular fatty acids triggers ER stress.^41^ Our results showed that cardiac tissues of db/db mice exhibit Bip and P‐eIF2α/eIF2α up‐regulation and the induction of CHOP, which suggests ER overstress. Exogenous H_2_S significantly reduced the expression of Bip and CHOP. These data were consistent with the results obtained with the cellular model. Our results suggested that exogenous H_2_S could attenuate ER stress. The occurrence of the UPR initiates the proteostasis system, such as ER chaperone‐assisted protein folding and ERAD, for the degradation of misfolded proteins.^42^ Hrd1, a multi‐transmembrane RING domain E3 ligase, plays a role in ER protein quality control. However, when maladaptive ER stress was induced, Hrd1 was not sufficient for driving the accumulation of misfolded proteins, which threatens cell survival. Our LC‐MS/MS results confirmed that the expression of Hrd1 was decreased in the hearts of db/db mice and that exogenous H_2_S could up‐regulate the expression of Hrd1.

Diacylglycerol acyltransferase (DGAT) enzymes, DGAT1 and DGAT2, catalysed the last step of LD formation and are responsible for the synthesis of TAG from diacylglycerol and fatty acyl CoAs.^43^ DGAT1 is localized to the ER and mediates the formation of initial LDs. In contrast, DGAT2 is localized in the ER and around LDs and is specialized in LDs.^44^ Our data demonstrated that the expression of DGAT1 and 2 was significantly increased in hearts of db/db mice. Some studies have confirmed that LDs increased in abundance where UPR is activated.^45^ The presence of 4‐PBA, an ER stress inhibitor, inhibits LD formation in ER under high glucose and lipid conditions. LD‐mediated ER quality control likely triggers the ERAD pathway. Therefore, Hrd1, as a major component of ERAD pathway, might be involved in regulating the formation of LDs. Our results suggested that Hrd1 could reduce the formation of LDs by modulating the ubiquitination level of DGAT1 and DGAT2.

H_2_S, as an active gasotransmitter, mediates many pathophysiological processes. Recent studies have shown that H_2_S can modify the target proteins via the process of S‐sulfhydration, a recently discovered post‐translational modification.^46^, ^47^ Cysteines in proteins can be oxidized to form reactive cysteines, such as cysteine sulfenic acid or glutathiolated cysteine, which have a low pKa and a positive electrostatic potential, and these reactive cysteines combine with sulphide anions to form cysteine persulphide.^48^, ^49^ Our results showed that the exposure of heart tissue and H9c2 cells to NaHS increased the S‐sulfhydration level of Hrd1. The overexpression of Hrd1‐C115A decreased the S‐sulfhydration level of Hrd1, inhibited the interaction between Hrd1 and DGAT1 and increased the number of LDs. These results suggested that H_2_S reduced LD formation by modulating the S‐sulfhydration level of Hrd1.

In summary, this study demonstrated that H_2_S increased S‐sulfhydration of Hrd1 at Cys115 to regulate the ubiquitination level of DGAT1 and 2 and thereby prevent the formation of LDs.

## 5 INNOVATION

**FIGURE 7:**
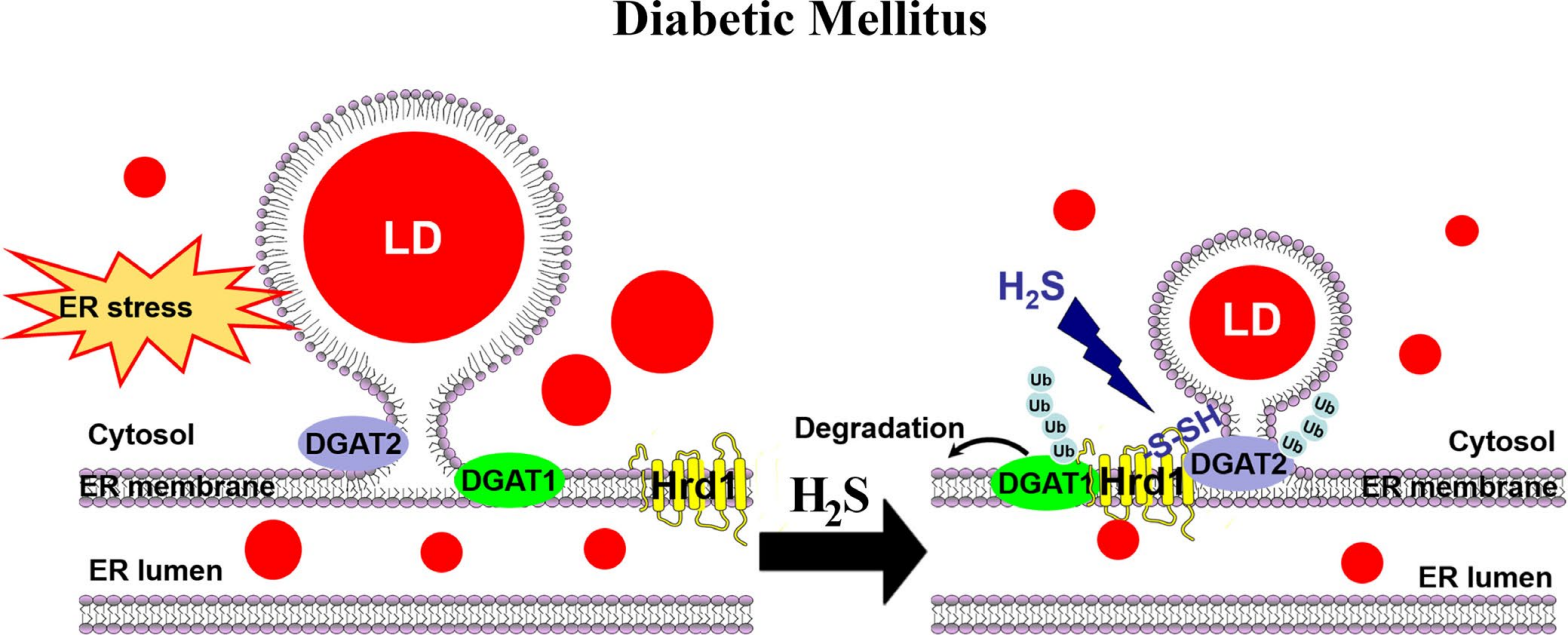
Model for the role of H_2_S in regulating of formation of lipid droplets in cardiac tissues

This study revealed the following: (a) H_2_S protect against diabetic cardiomyopathy by reducing the accumulation of LDs and (b) S‐sulfhydrated Hrd1 at cysteine 115 elevates the ubiquitination level of DGAT1 and 2 to inhibit the LD formation. These results uncover a new mechanism through which H_2_S regulates the formation of LDs to ameliorate diabetic cardiomyopathy (Figure 7).

## CONFLICT OF INTERESTS

The authors declare that they have no conflict of interests.

## AUTHOR CONTRIBUTIONS

**Yu Sun :** Investigation (equal); Writing‐original draft (equal). **Linxue Zhang:** Data curation (equal); Formal analysis (equal); Validation (equal). **Baoling Lu:** Writing‐original draft (equal). **Jingchen Wen:** Investigation (equal). **Mengyi Wang:** Investigation (equal); Validation (equal). **Shiwu Zhang:** Investigation (equal); Validation (equal). **Qianzhu Li:** Investigation (equal); Validation (equal). **Feng Shu:** Formal analysis (equal). **Fangping Lu:** Formal analysis (equal); Investigation (equal). **Ning Liu:** Formal analysis (equal); Investigation (equal). **Shuo Peng:** Formal analysis (equal); Investigation (equal). **Yajun Zhao :** Formal analysis (equal). **Shiyun Dong:** Formal analysis (equal). **Fanghao Lu :** Resources (equal); Writing‐original draft (equal); Writing‐review & editing (equal). **weihua zhang:** Resources (lead); Supervision (lead); Writing‐original draft (lead); Writing‐review & editing (lead). **Yan Wang:** Project administration (equal); Writing‐original draft (equal).

## Supporting information

Supplementary Material

## Data Availability

The data sets used and/or analysed in the current study are available from the corresponding author upon reasonable request.

## References

[1] Lee YJ , Woo SY , Ahn JH , Cho S , Kim SR . Health‐related quality of life in adults with metabolic syndrome: the Korea national health and nutrition examination survey, 2007–2008. Ann Nutr Metab. 2012;61(4):275‐280. 23208156 10.1159/000341494

[2] Nakamura M. , Sadoshima J. . Cardiomyopathy in obesity, insulin resistance and diabetes. J Physio. 2020;598 (14):2977‐2993. 10.1113/jp276747 30869158

[3] Bozkurt B , Aguilar D , Deswal A , et al. Contributory Risk and Management of Comorbidities of Hypertension, Obesity, Diabetes Mellitus, Hyperlipidemia, and Metabolic Syndrome in Chronic Heart Failure: A Scientific Statement From the American Heart Association. Circulation. 2016;134(23):e535‐e578. 27799274 10.1161/CIR.0000000000000450

[4] Kubler W , Kuhn H , Loogen F . Clinical classification of myocardiopathies. Dtsch Med Wochenschr. 1972;97(47):1834‐1836. 4673976

[5] Sharma S , Adrogue JV , Golfman L , et al. Intramyocardial lipid accumulation in the failing human heart resembles the lipotoxic rat heart. FASEB J. 2004;18(14):1692‐1700. 15522914 10.1096/fj.04-2263com

[6] Chong CR , Clarke K , Levelt E . Metabolic Remodeling in Diabetic Cardiomyopathy. Cardiovasc Res. 2017;113(4):422‐430. 28177068 10.1093/cvr/cvx018 PMC5412022

[7] Schilling JD , Mann DL . Diabetic cardiomyopathy: bench to bedside. Heart Fail Clin. 2012;8(4):619‐631. 22999244 10.1016/j.hfc.2012.06.007 PMC3457018

[8] Goldberg IJ , Reue K , Abumrad NA , et al. Deciphering the role of lipid droplets in cardiovascular disease: a report from the 2017 national heart, lung, and blood institute workshop. Circulation. 2018;138(3):305‐315. 30012703 10.1161/CIRCULATIONAHA.118.033704 PMC6056021

[9] Walther TC , Chung J , Farese RV Jr . Lipid droplet biogenesis. Annu Rev Cell Dev Biol. 2017;33:491‐510. 28793795 10.1146/annurev-cellbio-100616-060608 PMC6986389

[10] Cohen DE , Fisher EA . Lipoprotein metabolism, dyslipidemia, and nonalcoholic fatty liver disease. Semin Liver Dis. 2013;33(4):380‐388. 24222095 10.1055/s-0033-1358519 PMC3988578

[11] Pagliassotti MJ , Kim PY , Estrada AL , Stewart CM , Gentile CL . Endoplasmic reticulum stress in obesity and obesity‐related disorders: an expanded view. Metabolism. 2016;65(9):1238‐1246. 27506731 10.1016/j.metabol.2016.05.002 PMC4980576

[12] Qi L , Tsai B , Arvan P . New insights into the physiological role of endoplasmic reticulum‐associated degradation. Trends Cell Biol. 2017;27(6):430‐440. 28131647 10.1016/j.tcb.2016.12.002 PMC5440201

[13] Wei J , Yuan Y , Chen L , et al. ER‐associated ubiquitin ligase HRD1 programs liver metabolism by targeting multiple metabolic enzymes. Nat Commun. 2018;9(1):3659. 30201971 10.1038/s41467-018-06091-7 PMC6131148

[14] Doroudgar S , Völkers M , Thuerauf DJ , et al. Hrd1 and ER‐associated protein degradation, ERAD, are critical elements of the adaptive ER stress response in cardiac myocytes. Circ Res. 2015;117(6):536‐546. 26137860 10.1161/CIRCRESAHA.115.306993 PMC4670262

[15] Jarc E , Petan T . Lipid droplets and the management of cellular stress. Yale J Biol Med. 2019;92(3):435‐452. 31543707 PMC6747940

[16] Dalhaimer P . Lipid droplets in disease. Cells. 2019;8(9):974. 10.3390/cells8090974 PMC6770496 31454885

[17] Polhemus DJ , Lefer DJ . Emergence of hydrogen sulfide as an endogenous gaseous signaling molecule in cardiovascular disease. Circ Res. 2014;114(4):730‐737. 24526678 10.1161/CIRCRESAHA.114.300505 PMC3951140

[18] Predmore BL , Kondo K , Bhushan S , et al. The polysulfide diallyl trisulfide protects the ischemic myocardium by preservation of endogenous hydrogen sulfide and increasing nitric oxide bioavailability. Am J Physiol Heart Circ Physiol. 2012;302(11):H2410‐H2418. 22467307 10.1152/ajpheart.00044.2012 PMC3378306

[19] Kang J , Li Z , Organ CL , et al. pH‐controlled hydrogen sulfide release for myocardial ischemia‐reperfusion injury. J Am Chem Soc. 2016;138(20):6336‐6339. 27172143 10.1021/jacs.6b01373

[20] Li Z , Organ CL , Kang J , et al. Hydrogen sulfide attenuates renin angiotensin and aldosterone pathological signaling to preserve kidney function and improve exercise tolerance in heart failure. JACC Basic Transl Sci. 2018;3(6):796‐809. 30623139 10.1016/j.jacbts.2018.08.011 PMC6315048

[21] Whiteman M , Gooding KM , Whatmore JL , et al. Adiposity is a major determinant of plasma levels of the novel vasodilator hydrogen sulphide. Diabetologia. 2010;53(8):1722‐1726. 20414636 10.1007/s00125-010-1761-5

[22] Suzuki K , Olah G , Modis K , et al. Hydrogen sulfide replacement therapy protects the vascular endothelium in hyperglycemia by preserving mitochondrial function. Proc Natl Acad Sci USA. 2011;108(33):13829‐13834. 21808008 10.1073/pnas.1105121108 PMC3158211

[23] Ng HH , Yildiz GS , Ku JM , Miller AA , Woodman OL , Hart JL . Chronic NaHS treatment decreases oxidative stress and improves endothelial function in diabetic mice. Diab Vasc Dis Res. 2017;14(3):246‐253. 28467198 10.1177/1479164117692766

[24] Mustafa AK , Gadalla MM , Sen N , et al. H2S signals through protein S‐sulfhydration. Sci Signal. 2009;2(96):ra72. 19903941 10.1126/scisignal.2000464 PMC2998899

[25] Kang K , Zhao M , Jiang H , Tan G , Pan S , Sun X . Role of hydrogen sulfide in hepatic ischemia‐reperfusion‐induced injury in rats. Liver Transpl. 2009;15(10):1306‐1314. 19790158 10.1002/lt.21810

[26] Wang H , Lei M , Hsia RC , Sztalryd C . Analysis of lipid droplets in cardiac muscle. Methods Cell Biol. 2013;116:129‐149. 24099291 10.1016/B978-0-12-408051-5.00008-5 PMC7446730

[27] Sun Y , Tian Z , Liu N , et al. Exogenous H2S switches cardiac energy substrate metabolism by regulating SIRT3 expression in db/db mice. J Mol Med (Berl). 2018;96(3–4):281‐299. 29349500 10.1007/s00109-017-1616-3

[28] Schwanhäusser B , Busse D , Li NA , et al. Global quantification of mammalian gene expression control. Nature. 2011;473(7347):337‐342. 21593866 10.1038/nature10098

[29] de Hoon M , Imoto S , Nolan J , Miyano S . Open source clustering software. Bioinformatics. 2004;20(9):1453‐1454. 10.1093/bioinformatics/bth078 14871861

[30] Sun X , Zhao D , Lu F , et al. Hydrogen sulfide regulates muscle RING finger‐1 protein S‐sulfhydration at Cys(44) to prevent cardiac structural damage in diabetic cardiomyopathy. Br J Pharmacol. 2019;177(4):836‐856. 30734268 10.1111/bph.14601 PMC7024715

[31] Meng G , Zhao S , Xie L , Han Y , Ji Y . Protein S‐sulfhydration by hydrogen sulfide in cardiovascular system. Br J Pharmacol. 2018;175(8):1146‐1156. 28432761 10.1111/bph.13825 PMC5866969

[32] Yang F , Yu X , Li T , et al. Exogenous H2S regulates endoplasmic reticulum‐mitochondria cross‐talk to inhibit apoptotic pathways in STZ‐induced type I diabetes. Am J Physiol Endocrinol Metab. 2017;312(3):E190‐E203. 27998959 10.1152/ajpendo.00196.2016

[33] Rambold AS , Cohen S , Lippincott‐Schwartz J . Fatty acid trafficking in starved cells: regulation by lipid droplet lipolysis, autophagy, and mitochondrial fusion dynamics. Dev Cell. 2015;32(6):678‐692. 25752962 10.1016/j.devcel.2015.01.029 PMC4375018

[34] Borradaile NM , Han X , Harp JD , Gale SE , Ory DS , Schaffer JE . Disruption of endoplasmic reticulum structure and integrity in lipotoxic cell death. J Lipid Res. 2006;47(12):2726‐2737. 16960261 10.1194/jlr.M600299-JLR200

[35] Welte MA , Gould AP . Lipid droplet functions beyond energy storage. Biochim Biophys Acta Mol Cell Biol Lipids. 2017;1862(10):1260‐1272. 10.1016/j.bbalip.2017.07.006 28735096 PMC5595650

[36] Greiner R , Palinkas Z , Basell K , et al. Polysulfides link H2S to protein thiol oxidation. Antioxid Redox Signal. 2013;19(15):1749‐1765. 23646934 10.1089/ars.2012.5041 PMC3837443

[37] Jia G , Hill MA , Sowers JR . Diabetic cardiomyopathy: an update of mechanisms contributing to this clinical entity. Circ Res. 2018;122(4):624‐638. 29449364 10.1161/CIRCRESAHA.117.311586 PMC5819359

[38] Osumi T , Kuramoto K . Heart lipid droplets and lipid droplet‐binding proteins: biochemistry, physiology, and pathology. Exp Cell Res. 2016;340(2):198‐204. 26524506 10.1016/j.yexcr.2015.10.031

[39] Ljubkovic M , Gressette M , Bulat C , et al. Disturbed fatty acid oxidation, endoplasmic reticulum stress, and apoptosis in left ventricle of patients with type 2 diabetes. Diabetes. 2019;68(10):1924‐1933. 31391173 10.2337/db19-0423

[40] Dai B , Li H , Fan J , et al. MiR‐21 protected against diabetic cardiomyopathy induced diastolic dysfunction by targeting gelsolin. Cardiovasc Diabetol. 2018;17(1):123. 30180843 10.1186/s12933-018-0767-z PMC6122727

[41] Park M , Sabetski A , Kwan Chan Y , Turdi S , Sweeney G . Palmitate induces ER stress and autophagy in H9c2 cells: implications for apoptosis and adiponectin resistance. J Cell Physiol. 2015;230(3):630‐639. 25164368 10.1002/jcp.24781

[42] Hwang J , Qi L . Quality control in the endoplasmic reticulum: crosstalk between ERAD and UPR pathways. Trends Biochem Sci. 2018;43(8):593‐605. 30056836 10.1016/j.tibs.2018.06.005 PMC6327314

[43] Bhatt‐Wessel B , Jordan TW , Miller JH , Peng L . Role of DGAT enzymes in triacylglycerol metabolism. Arch Biochem Biophys. 2018;655:1‐11. 30077544 10.1016/j.abb.2018.08.001

[44] Irshad Z , Chmel N , Adya R , Zammit VA . Hepatic VLDL secretion: DGAT1 determines particle size but not particle number, which can be supported entirely by DGAT2. J Lipid Res. 2019;60(1):111‐120. 30397187 10.1194/jlr.M089300 PMC6314258

[45] Lee JS , Mendez R , Heng HH , Yang ZQ , Zhang K . Pharmacological ER stress promotes hepatic lipogenesis and lipid droplet formation. Am J Transl Res. 2012;4(1):102‐113. 22347525 PMC3276380

[46] Wu D , Hu Q , Tan B , Rose P , Zhu D , Zhu YZ . Amelioration of mitochondrial dysfunction in heart failure through S‐sulfhydration of Ca(2+)/calmodulin‐dependent protein kinase II. Redox Biol. 2018;19:250‐262. 30195191 10.1016/j.redox.2018.08.008 PMC6128039

[47] Yang G , Zhao K , Ju Y , et al. Hydrogen sulfide protects against cellular senescence via S‐sulfhydration of Keap1 and activation of Nrf2. Antioxid Redox Signal. 2013;18(15):1906‐1919. 23176571 10.1089/ars.2012.4645

[48] Krishnan N , Fu C , Pappin DJ , Tonks NK . H2S‐Induced sulfhydration of the phosphatase PTP1B and its role in the endoplasmic reticulum stress response. Sci Signal. 2011;4(203):ra86. 22169477 10.1126/scisignal.2002329 PMC3328411

[49] Yadav PK , Martinov M , Vitvitsky V , et al. Biosynthesis and reactivity of cysteine persulfides in signaling. J Am Chem Soc. 2016;138(1):289‐299. 26667407 10.1021/jacs.5b10494 PMC4795164

