# Supplementary material for: Hydrogen sulphide reduced the accumulation of lipid droplets in cardiac tissues of db/db mice via Hrd1 S‐sulfhydration
Source: J Cell Mol Med. 2021 Sep 25;25(19):9154–67. doi: 10.1111/jcmm.16781 (PMC8500968; doi:10.1111/jcmm.16781)
Supplement: Supplementary file 1 — Supplementary Material [file JCMM-25-9154-s001.doc]

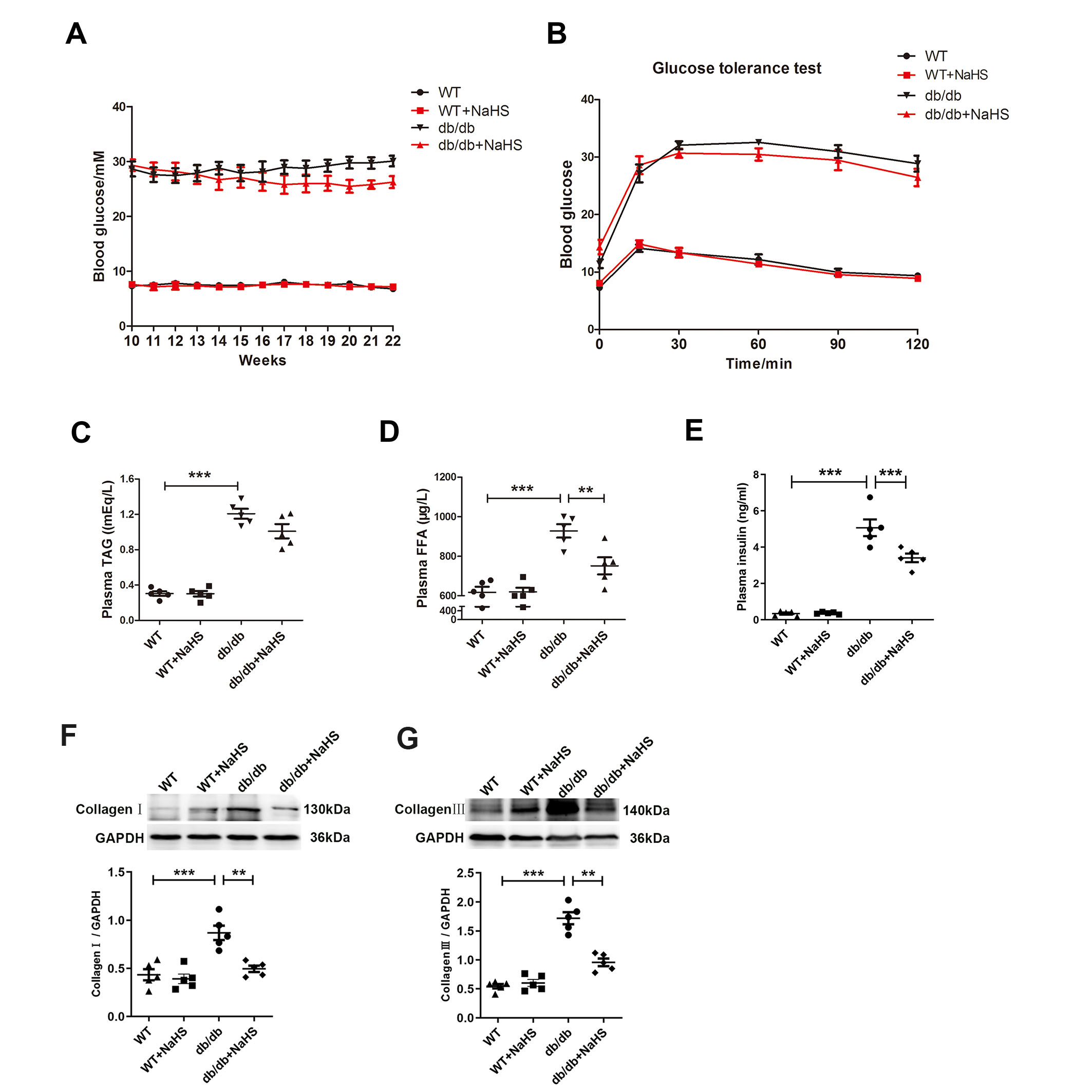
**Supplementary**


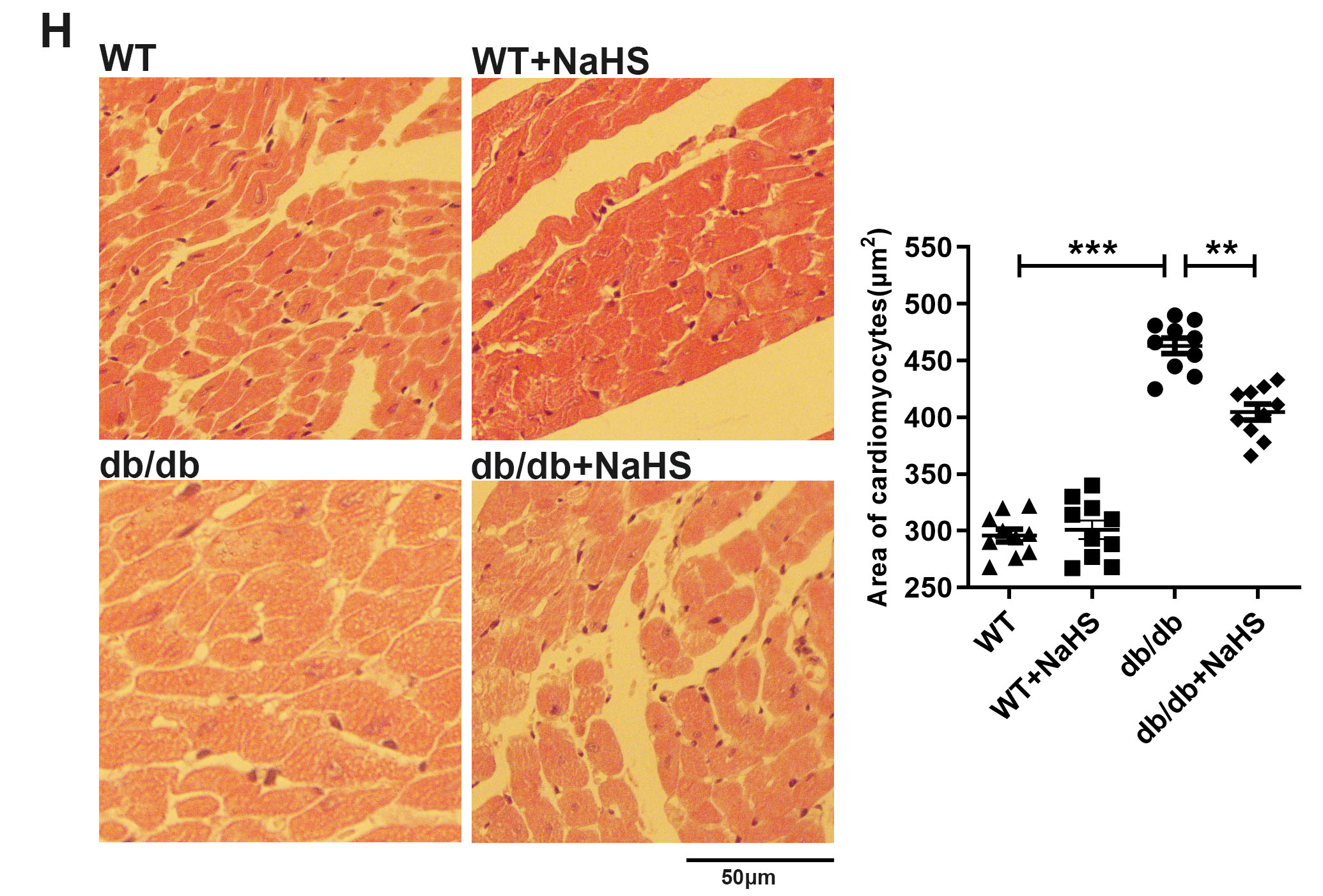


**Figure S1.** **General indexes of db/db mice.** The db/db mice and its wild-type mice were injected with NaHS (4.8 mg/kg) or saline by intraperitoneal injection every 2 days for 12 weeks. **(A)** Glucose level. **(B)** Glucose tolerance test. **(C)** Plasma triglyceride content. **(D)** Plasma free fatty acid content. **(E)** Plasma insulin content. Western blot analyzed the expression of collagen I **(F)** and collagen Ⅲ **(G)** in cardiac tissues of mice. The values represent the means ± SE. ***P*＜0.01, ****P*＜0.001, n=5. **(H)** The morphology of cardiac tissues in db/db micce were detected by HE staining. The values represent the means ± SE. ***P*＜0.01, ****P*＜0.001, an asterisk indicates a significant difference based on n=10 myocytes from five independent biological replicates.


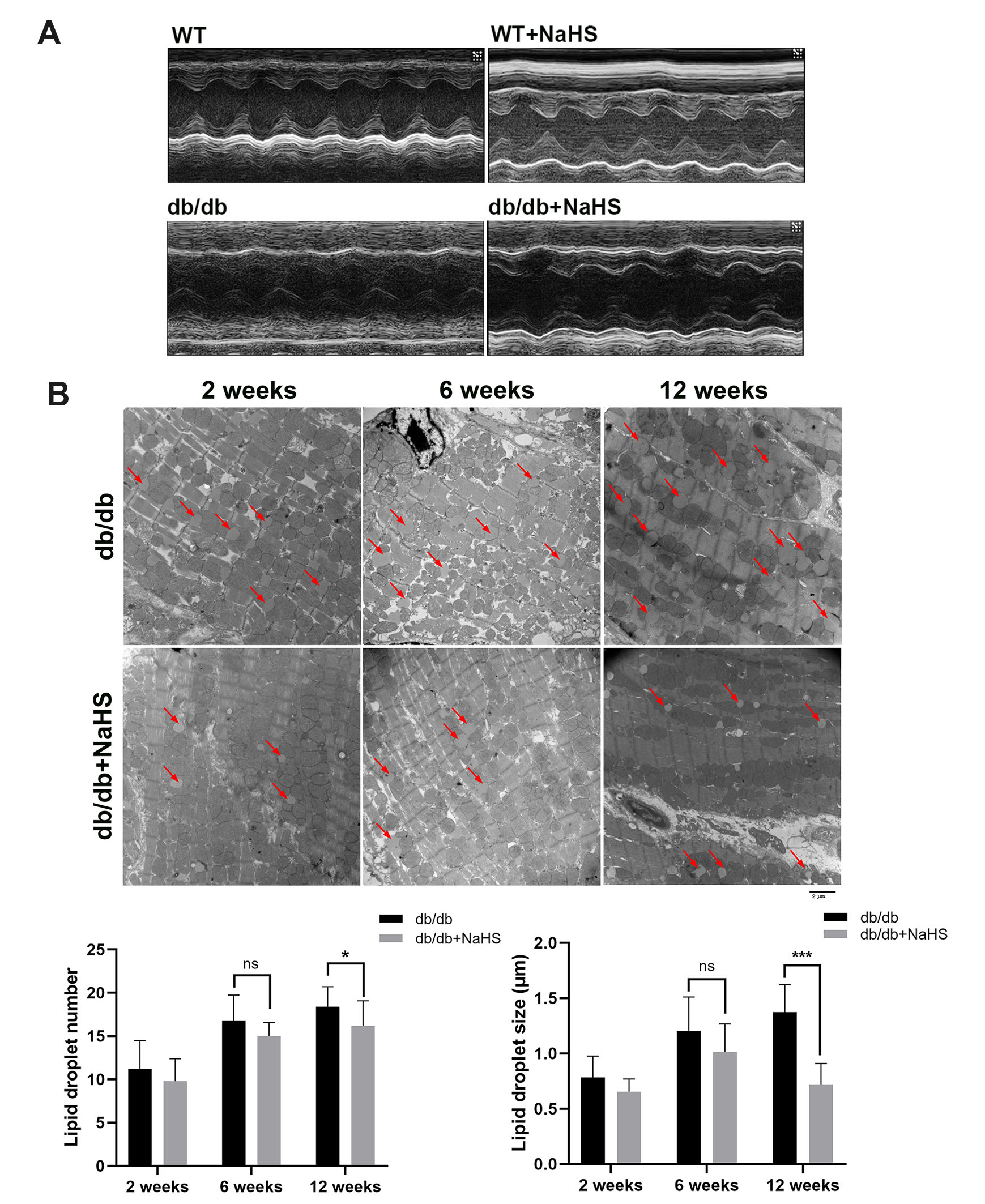


**Figure S2. (A)** M-mode echocardiography. **(B)** The ultrastructure of the db/db mouse myocardium after treatment with NaHS for different times was observed by TEM. Lipid droplets were indicated by red arrows. An asterisk indicates a significant difference (**P*< 0.05, ****P*< 0.001, *t* test) based on n=10 lipid droplets from five independent biological replicates.


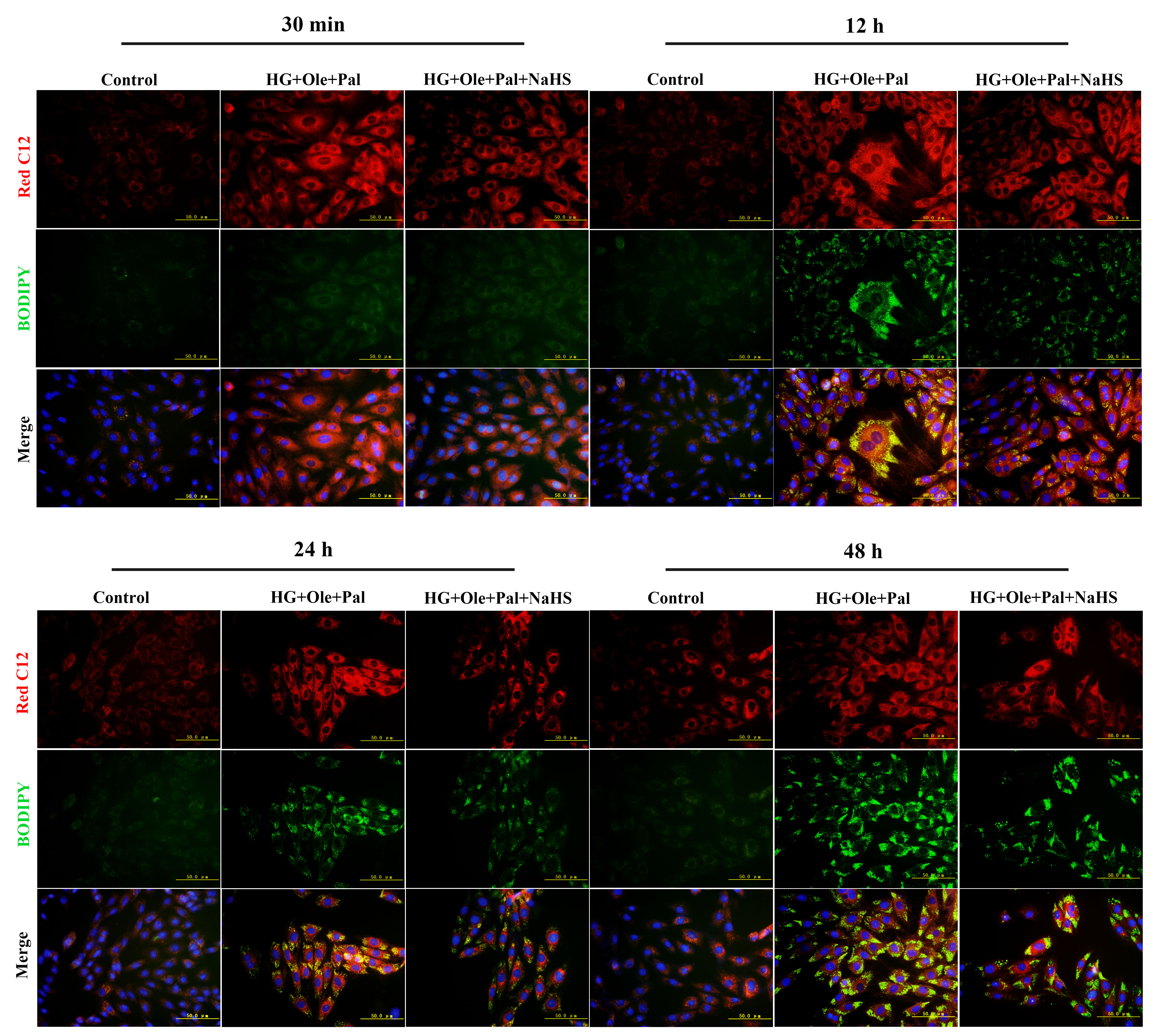


**Figure S3. Detection of droplets in H9c2 cells using the fluorescence probe BODIPY493/503 or BODIPY 558/469 C12.**


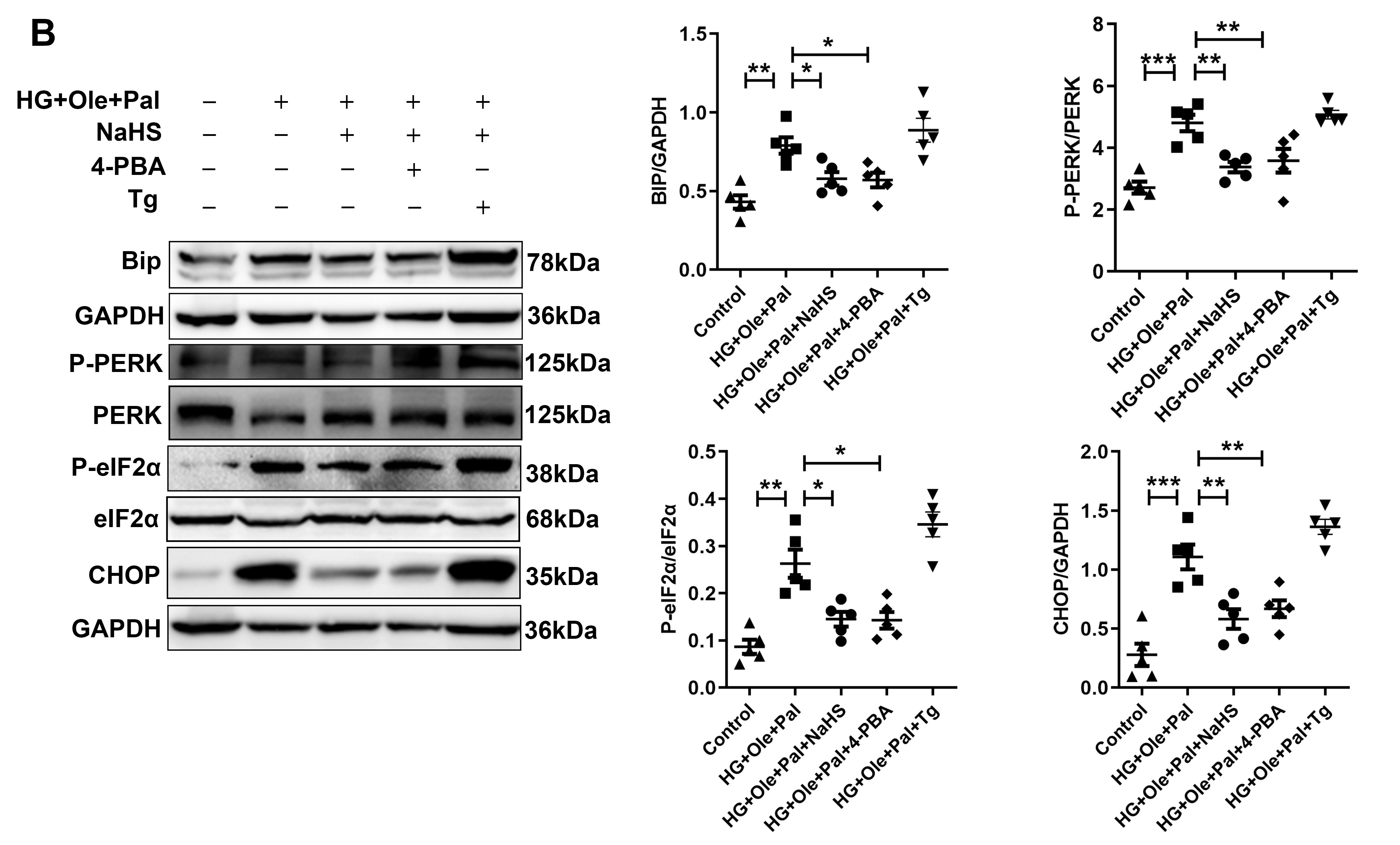

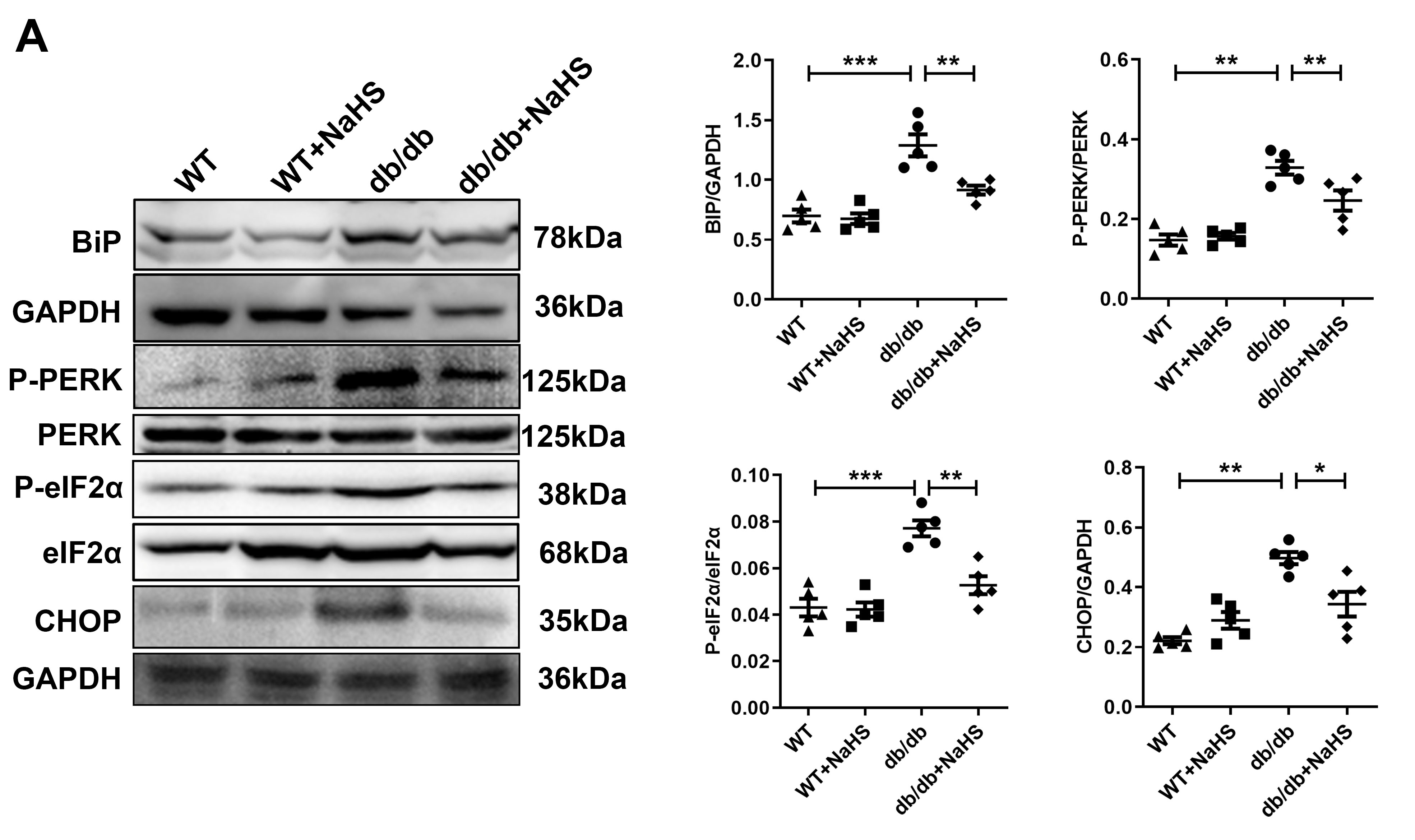


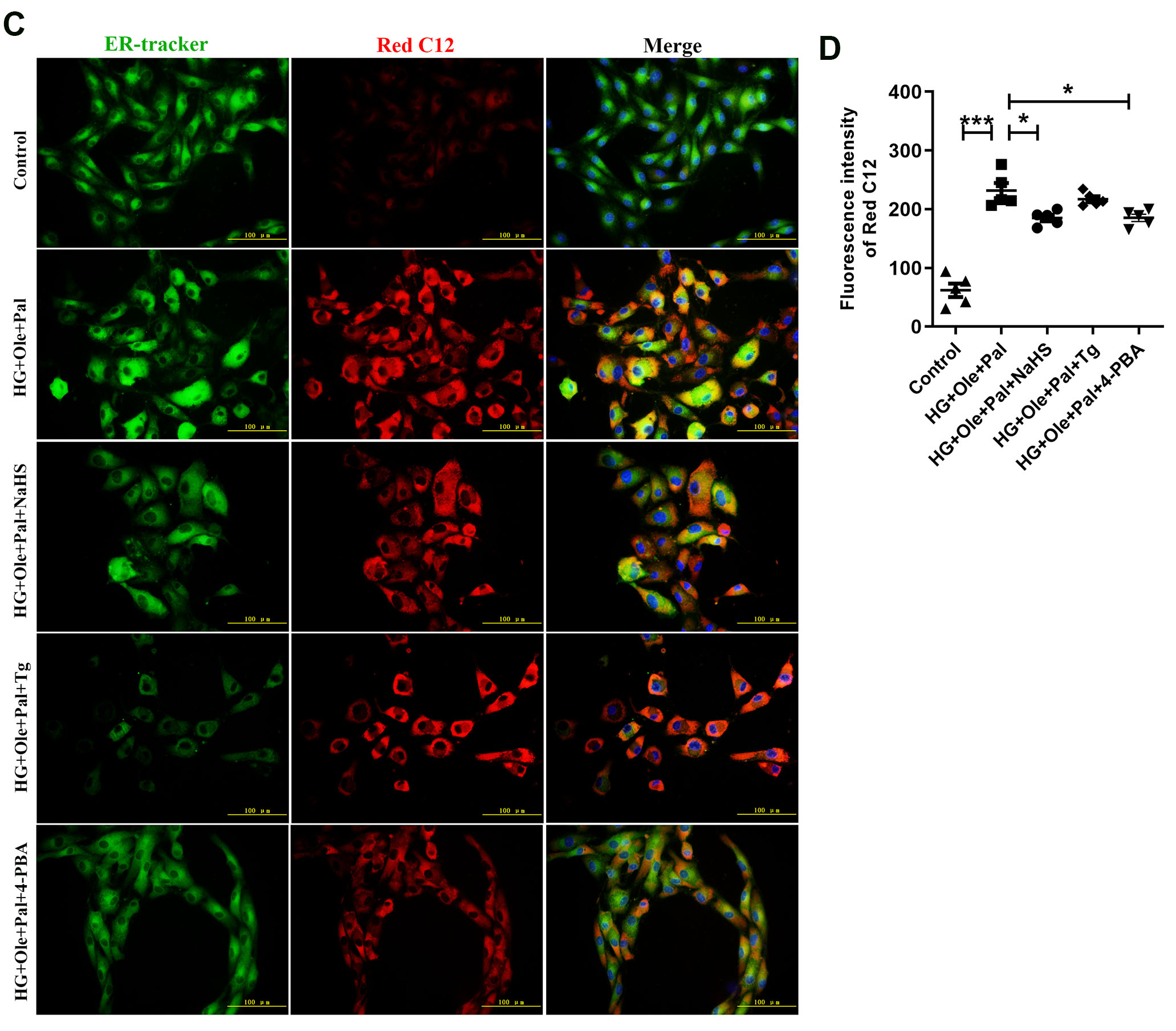
**Figure S4. Exogenous H_2_S inhibits LD formation by attenuating ER stress.** **(A)** Expression of ER stress-related proteins in cardiac tissues. **(B)** Expression of ER stress-related in H9c2 cells. **(C)**The colocalization between lipid droplet and ER was detected by immunofluorescence. **(D)** Quantification of fluorescence intensity statistics. The values represent the means ± SE; n=5.**P*＜0.05, ***P*＜0.01, ****P*＜0.001.


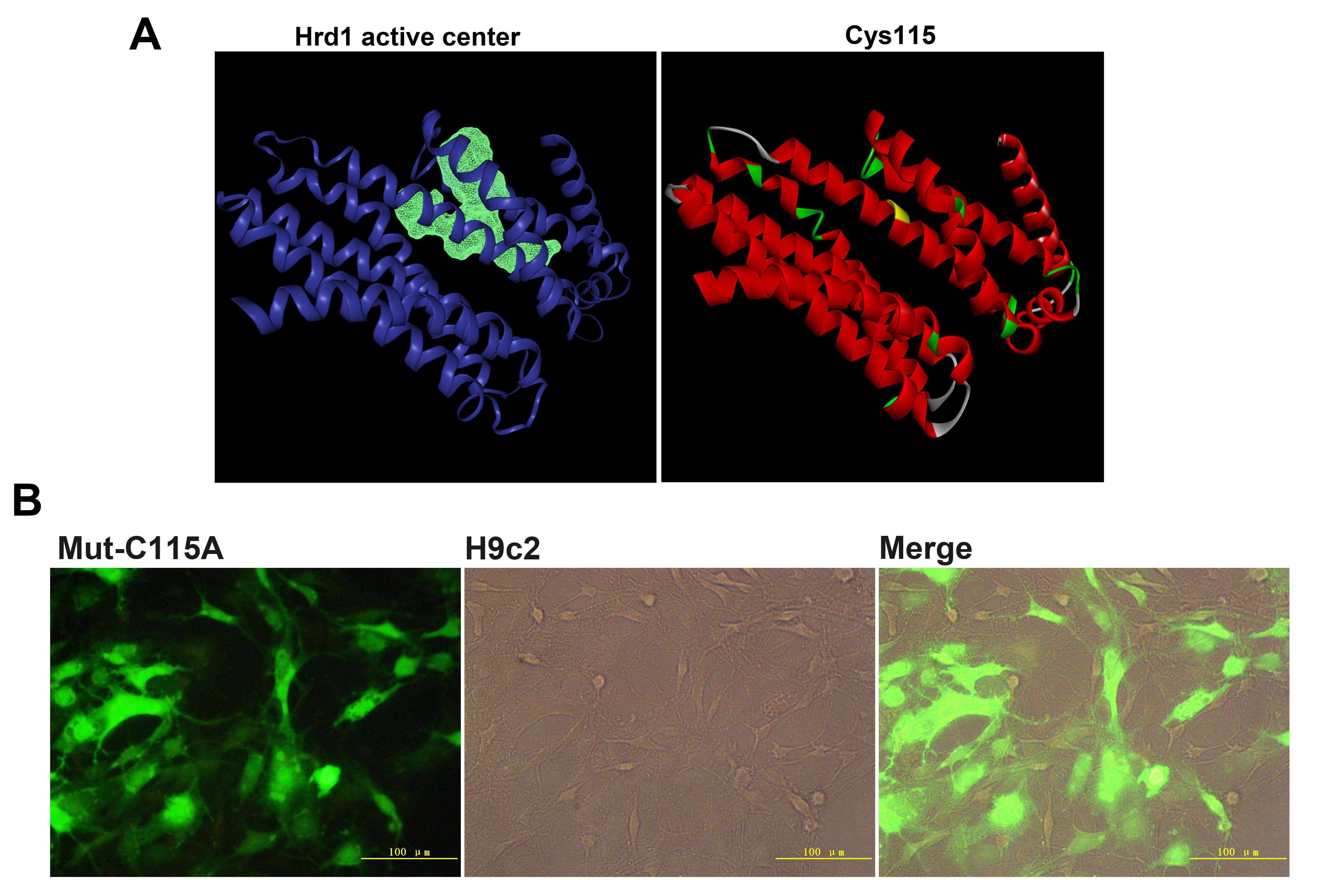


**Figure S5. (A)**Prediction of the active centre of Hrd1 using a computational method. **(B)** Hrd1 mutated at Cys115 was transfected into H9c2 cells for 48 hours. The GFP (green fluorescence) were shown in the left picture, and the H9c2 cells of the same location was detected in the middle picture, the merge was shown in the right picture.

**Table S1. Comparative proteomic analysis of lipid droplet in cardiac tissues from db/db and NaHS-treated db/db mice.**
